# Sorcin can trigger pancreatic cancer-associated new-onset diabetes through the secretion of inflammatory cytokines such as serpin E1 and CCL5

**DOI:** 10.1038/s12276-024-01346-4

**Published:** 2024-11-08

**Authors:** Jiali Gong, Xiawei Li, Zengyu Feng, Jianyao Lou, Kaiyue Pu, Yongji Sun, Sien Hu, Yizhao Zhou, Tianyu Song, Meihua Shangguan, Kai Zhang, Wenjie Lu, Xin Dong, Jian Wu, Hong Zhu, Qiaojun He, Hongxia Xu, Yulian Wu

**Affiliations:** 1 Second Affiliated Hospital, Zhejiang University School of Medicine, Hangzhou, Zhejiang China; 2 Key Laboratory of Cancer Prevention and Intervention, China National Ministry of Education, Cancer Institute, Second Affiliated Hospital, Zhejiang University School of Medicine, Hangzhou, Zhejiang China; 3 Cancer Center, Zhejiang University, Hangzhou, Zhejiang China; 4 Department of Surgery, Fourth Affiliated Hospital, Zhejiang University School of Medicine, Yiwu, Zhejiang China; 5 Harvard T.H. Chan School of Public Health, Harvard University, Boston, MA USA; 6 School of Public Health and Eye Center The Second Affiliated Hospital, Zhejiang University, Hangzhou, China; 7 School of Public Health, Zhejiang University School of Medicine, Hangzhou, Zhejiang China; 8 Institute of Wenzhou, Zhejiang University, Wenzhou, Zhejiang China; 9 Zhejiang Province Key Laboratory of Anti-Cancer Drug Research, College of Pharmaceutical Sciences, Zhejiang University, Hangzhou, Zhejiang China; 10 Center for Drug Safety Evaluation and Research of Zhejiang University, Hangzhou, Zhejiang China; 11 Innovation Institute for Artificial Intelligence in Medicine and Liangzhu Laboratory, Zhejiang University School of Medicine, Zhejiang University, Hangzhou, China

**Keywords:** Diagnostic markers, Cancer genetics, Tumour biomarkers

## Abstract

A rise in blood glucose is an early warning sign of underlying pancreatic cancer (PC) and may be an indicator of genetic events in PC progression. However, there is still a lack of mechanistic research on pancreatic cancer-associated new-onset diabetes (PCAND). In the present study, we identified a gene *SRI*, which possesses a SNP with the potential to distinguish PCAND and Type 2 diabetes mellitus (T2DM), by machine learning on the basis of the UK Biobank database. In vitro and in vivo, sorcin overexpression induced pancreatic β-cell dysfunction. Sorcin can form a positive feedback loop with STAT3 to increase the transcription of serpin E1 and CCL5, which may directly induce β-cell dysfunction. In 88 biopsies, the expression of sorcin was elevated in PC tissues, especially in PCAND samples. Furthermore, plasma serpin E1 levels are higher in peripheral blood samples from PCAND patients than in those from T2DM patients. In conclusion, sorcin may be the key driver in PCAND, and further study on the sorcin-STAT3-serpin E1/CCL5 signaling axis may help us better understand the pathogenesis of PCAND and identify potential biomarkers.

## Sorcin Identified as Key Driver in Pancreatic Cancer Diabetes

Pancreatic cancer, a lethal disease with a low survival rate, can be better managed with early detection. Researchers investigated the connection between pancreatic cancer and newly developed diabetes, pinpointing the SRI gene and its protein, sorcin, as crucial elements. They used machine learning to examine genetic data from the UK Biobank, focusing on Single Nucleotide Polymorphisms to differentiate between diabetes associated with pancreatic cancer and type 2 diabetes. The research included lab and live experiments to understand how sorcin impacts pancreatic β-cells and contributes to diabetes in pancreatic cancer patients. Researchers believe that focusing on the sorcin-STAT3 pathway could provide new possibilities for early diagnosis and treatment of pancreatic cancer. This study is a significant advancement in understanding the molecular links between pancreatic cancer and diabetes, potentially leading to earlier detection and better patient outcomes. This summary was initially drafted using artificial intelligence, then revised and fact-checked by the author.

## Introduction

Pancreatic cancer (PC) is a highly fatal disease with a 5-year cumulative survival rate of approximately 10% in the USA^1,2^. Early diagnosis of PC at a resectable stage provides more treatment options and substantially improves patient survival^3^. Previous studies of pancreatic tumorigenesis have suggested that mutations in PC driver genes occur in a specific order; activating mutations in *KRAS* are present in low-grade pancreatic intraepithelial neoplasia (PanIN-1) lesions^4^ (94.1% mutation rate^5^), and inactivating mutations in *CDKN2A* (17.0%), *TP53* (63.9%) and *SMAD4* (20.8%) occur thereafter and are found in transformed PanIN-2 and PanIN-3 lesions^6,7^. However, owing to the long duration PanIN-PC evolution and the lack of specific marker gene mutations^8^, related early diagnostic strategies have not achieved significant clinical benefits.

New-onset diabetes, especially in individuals aged over 50 years, has been identified as an early warning sign of underlying PC. A case‒control study revealed that, on average, patients with pancreatic ductal adenocarcinoma (PDAC) develop hyperglycemia 36 to 30 months before their tumor diagnosis^9^, presenting a potential window of opportunity for early detection. Distinguishing new-onset diabetes from the more prevalent type 2 diabetes mellitus (T2DM) is a prerequisite for targeted screening of this high-risk population. A recent study by Bao et al. suggested that pancreatic cancer-associated new-onset diabetes (PCAND) is characterized primarily by reduced insulin secretory capacity resulting from β-cell dysfunction^10^. Insulin resistance, though also present in PCAND patients^11^, appears to be less severe than that observed in patients with T2DM^10^. Recent studies have also identified a growing list of biomarkers associated with PCAND, including connexin-26^12^, vanin-1 (VNN-1) and matrix metalloproteinase 9 (MMP-9)^13,14^, galectin-3 and S100A9^15^, S-100A8 N-terminal peptide^16^, amylin^17^, the glucagon/insulin ratio^18^, insulin gene promoter polymorphisms^19^, adrenomedullin^20^, islet amyloid polypeptide (IAPP)^21^, fatty acid binding protein-1 (FABP-1)^22^ and insulin-like growth factor-I^23^. However, the mechanistic link between PC and the pathogenesis of new-onset diabetes remains largely unclear. The main challenge now is identifying the 1% of PCAND patients from common T2DM patients in the new-onset diabetes population^24–26^.

To identify the key regulator(s) involved in PCAND pathogenesis, we employed machine learning techniques to identify single nucleotide polymorphism (SNP) loci and their associated genes that possess discriminatory power in distinguishing between PCAND and T2DM. Finally, we identified *SRI* gene, which encodes a protein named sorcin (soluble resistance-related calcium binding protein)^27^. Interestingly, we found that sorcin was significantly overexpressed in tumor samples from PDAC patients, especially in PCAND patients. In vitro *and* in vivo, we found that sorcin overexpression can impair pancreatic β-cells. We showed that sorcin forms a positive feedback loop with STAT3 and activates the transcription of inflammatory factors, such as CCL5 and serpin E1. Finally, we preliminarily confirmed the potential of *SRI* and its downstream serpin E1 in distinguishing PCAND from T2DM on the basis of an online database and small clinical cohorts.

## Materials and methods

### UK Biobank Database Study Design and population

**Fig. 1:**
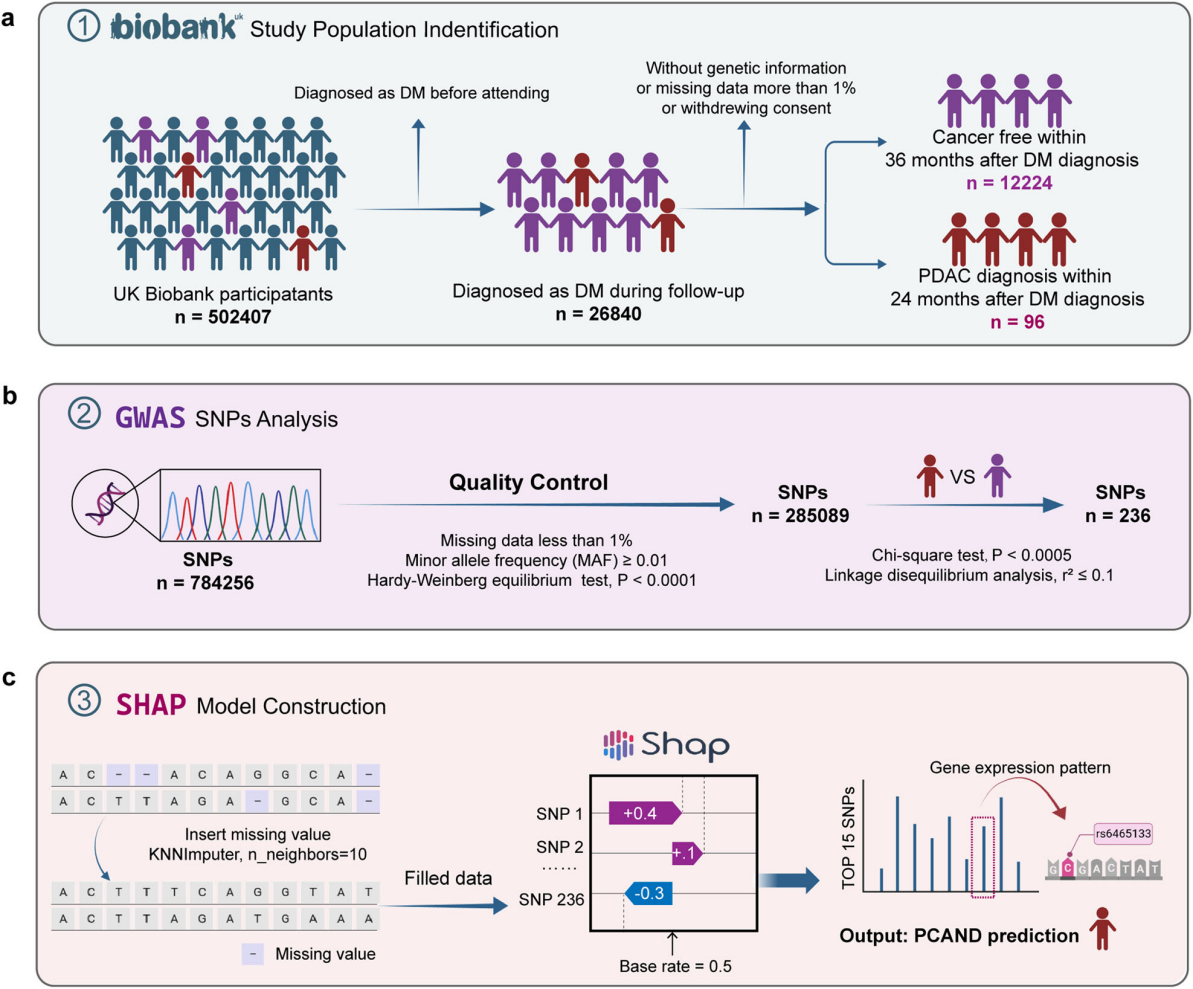
Research content and technique flow chart. **a** The procedure of population screening and partitioning into PCAND and pure T2DM cohorts in the UK Biobank. **b** The procedure of GWAS analysis. The quality of the genetic data was controlled by the Hardy‒Weinberg equilibrium (HWE) test (*P* values less than 0.0001). SNP loci with a *P* value < 0.0005 were identified as potentially significant loci between T2DM and PCAND. **c** The procedure of model construction via machine learning. SNPs exhibiting larger absolute Shapley additive explanation (SHAP) values are more important in individual predictions.

The UK Biobank is an ongoing project to demonstrate the successful collection and sharing of linked genetic, physical and clinical information at the population scale. Extensive genetic and clinical data have been collected for approximately 500,000 volunteers across the United Kingdom^28^. We identified patients with cancer using the International Classification of Diseases codes (version 10, ICD-10) that were recorded in the national cancer registry on the basis of hospital admissions and causes of death. T2DM cases were defined as having an ICD-10 code of E11.X. Only cases in which the individuals did not have T2DM or cancer at the date of the attending assessment center were included in this research and subsequently followed up for incident T2DM and PDAC events. The participants were then split into groups according to the following criteria: PCAND if diagnosed with PDAC within 24 months after the diagnosis of T2DM and T2DM if no cancer occurred during the follow-up, which was longer than 36 months after the diagnosis of T2DM. The specific study screening flow chart is presented in Fig. 1a. The current research was conducted via the UK Biobank Resource under Application 91799.

### Quality control of genetic data and GWAS analysis

Genome-wide genetic data are available for 488,000 participants from the UK Biobank. We utilized genotype data from this dataset, which can be accessed at this link (https://biobank.ndph.ox.ac.uk/showcase/label.cgi?id=263). To ensure data quality, we first extracted variants with less than 1% missing data. We subsequently extracted individuals with less than 1% missing data and extracted variants with a minor allele frequency (MAF) ≥ 0.01. Next, we conducted a Hardy‒Weinberg equilibrium (HWE) test and calculated the *P* values for all the SNPs, extracting those with HWE *P* values less than 0.0001. Ultimately, 285,089 SNPs and 12,320 individual samples were selected for subsequent GWAS, and the chi-square test was used as the analytical method. We considered SNP loci with a *P* value < 0.0005 as potentially significant loci in differentiating PCAND and T2DM and obtained 287 SNPs. We subsequently conducted linkage disequilibrium (LD) analysis. We performed LD pruning to select only one representative SNP from each block. SNP pairs were considered independent if their correlation (r^2^) was less than or equal to 0.1. After LD analysis, a total of 236 SNPs were selected. All data analyses were performed via PLINK 1.9 software. The flow chart is presented in Fig. 1b.

### Model construction

To further select meaningful SNPs, we assessed the efficacy of SNPs in differentiating PCAND from T2DM. We presumed that missing data were distributed randomly and adopted the K-nearest neighbors imputer algorithm (KNNImputer, n_neighbors=10) to manage the null values in the dataset. Subsequent to data imputation, the Shapley additive explanation (SHAP) values^29^ were leveraged to gain a deeper understanding of the significance of individual SNPs. Notably, SNPs exhibiting higher absolute SHAP values had greater value in individual predictions. In this study, SHAP values were computed via a logistic regression model. An evaluation of the relative values revealed that the SHAP value showed a significant drop around 0.35; the top 15 SNPs were selected, and the corresponding genes were identified. Using the Gene Expression Profiling Interactive Analysis (GEPIA) website (http://gepia.cancer-pku.cn/), we investigated the differential expression of these genes between pancreatic cancer and normal tissues and assessed the impact of these genes on pancreatic cancer prognosis. The flow chart is presented in Fig. 1c.

### Cell culture

The AsPC-1, PANC-1, CFPAC-1, BxPC-3, Mia Paca-2, HPDE6, PANC-02, HEK293T and MIN6 cell lines were purchased from the American Type Culture Collection (ATCC). PANC-1, CFPAC-1, Mia Paca-2, HPDE6 and PANC-02 cells were cultured in DMEM (Gibco, USA), and ASPC-1 and BxPC-3 cells were maintained in RPMI-1640 medium (Gibco, USA). MIN6 cells were cultured in RPMI-1640 medium supplemented with 50 μM β-mercaptoethanol (Cienry, Zhejiang, China). Both DMEM and RPMI-1640 media were supplemented with 10% FBS (YEASEN, Shanghai, China) and 100 units/mL penicillin and streptomycin (Cienry, Zhejiang, China). All the cells were cultured in a humidified incubator at 37 °C in a 5% CO_2_ atmosphere. The cells were passaged when 80–90% confluence was reached, and the media were changed every 2 days.

### Human cytokine array

A membrane-based antibody array (Proteome Profiler Human Cytokine Array Kit, R&D Systems, ARY005B) was used to profile 36 soluble proteins, mostly cytokines and chemokines, in the conditioned medium from PANC-1 cells transfected with either *SRI*-siRNA or NC-siRNA. The complete list of proteins represented in this antibody array can be found on the manufacturer’s website (https://www.rndsystems.com/products/proteome-profiler-human-cytokine-array-kit_ary005b).

### Animal study design

To verify the effect of *SRI* expression in PC cells on islet function in vivo, four- to six-week-old female nude mice (*n* = 24) were randomly divided into four groups. These mice received subcutaneous injections of pancreatic cancer cell lines stably transduced with lentivirus (pCDH-*ovSRI*, pCDH-*shSRI* and pCDH-*NC*; 1×10^6^ cells in 100 μL of PBS for each line) or PBS (100 μL for each line). Blood glucose concentrations and body weights were measured every 4 days from the day after the first intraperitoneal injection. On the 24th day after subcutaneous injection, the nude mice were fasted for 24 h and then sacrificed, after which their peripheral blood, tumor tissue and pancreatic tissue were collected. Peripheral blood was used to measure fasting blood glucose and fasting insulin levels, and pancreatic tissue was used for immunofluorescence detection of insulin levels.

To verify the damaging effects of the cytokines serpin E1 and CCL5 on islets in vivo, four- to six-week-old female nude mice (*n* = 30) were randomly divided into six groups. These mice received subcutaneous injections of pancreatic cancer cell lines stably transduced with lentivirus (pCDH-*shSRI* and pCDH-*NC*, 1×10^6^ cells in 100 μL of PBS for each one) and received intratumoral injections of the cytokines serpin E1 and CCL5 (100 nM for each, recombinant protein from MedChemExpress, USA) or PBS (100 μL for each). The monitoring and sample testing methods used were the same as those described above. All animal experiments were approved by the ethics committee of ZheJiang University, and the methods for in vivo studies were carried out in accordance with the approved guidelines.

### Clinical study design and population

Eighty-eight PDAC biopsies, consisting of samples from 32 patients without diabetes (pure PC), 28 patients with new-onset diabetes (PCAND, with diabetes diagnosed 24 months before the diagnosis of PDAC^30^), and 28 patients with long-standing T2DM (PC + T2DM, with diabetes diagnosed > 24 months before the diagnosis of PDAC), were obtained at the Second Affiliated Hospital of Zhejiang University between January 2013 and December 2017. The diagnostic criteria for T2DM were in accordance with the American Diabetes Association^31^. All the pancreatic biopsies were classified according to the American Joint Committee on Cancer (AJCC) Staging Manual, 6th Edition. Twenty-one peripheral blood samples, consisting of 8 PCAND cases and 13 T2DM cases, were collected between January 2018 and January 2021. This study was approved by the ethics committees of Zhejiang University. This study was approved by the ethics committees of the Second Affiliated Hospital of Zhejiang University (Approval Number: I2019001590).

### Measurement of cytokine levels

Peripheral blood samples from pancreatic cancer patients with new-onset diabetes (*n* = 8) and type 2 diabetes patients (*n* = 13) were collected between January 2018 and January 2021 at the Second Affiliated Hospital of Zhejiang University. After anticoagulant treatment and centrifugation at 3000 rpm for 10 min, the plasma concentrations of the cytokines CCL5 and serpin E1 were measured with an ELISA kit (CUSABIO, Wuhan, China) according to the manufacturer’s instructions.

### Statistical analysis

The data were acquired from at least three independent experiments and are presented as the means ± SDs. All the statistical analyses were performed in GraphPad Prism version 8.0.2. Unpaired Student’s t test was used for comparisons between two groups, and one-way ANOVA was used for comparisons among multiple groups. Kaplan–Meier curves of overall survival were compared via the log-rank test. Correlation coefficients were calculated via the Pearson method. F values indicated variations between groups. The higher the F value is, the greater the difference between groups; the significance of differences was assessed via the *P* value, and *P* < 0.05 was considered to indicate statistical significance.

Please see the Supplementary Information for details on the materials and processes used in this study.

## Results

### The SNP rs6465133 in *SRI* has the potential to distinguish PCAND from T2DM via machine learning

**Fig. 2:**
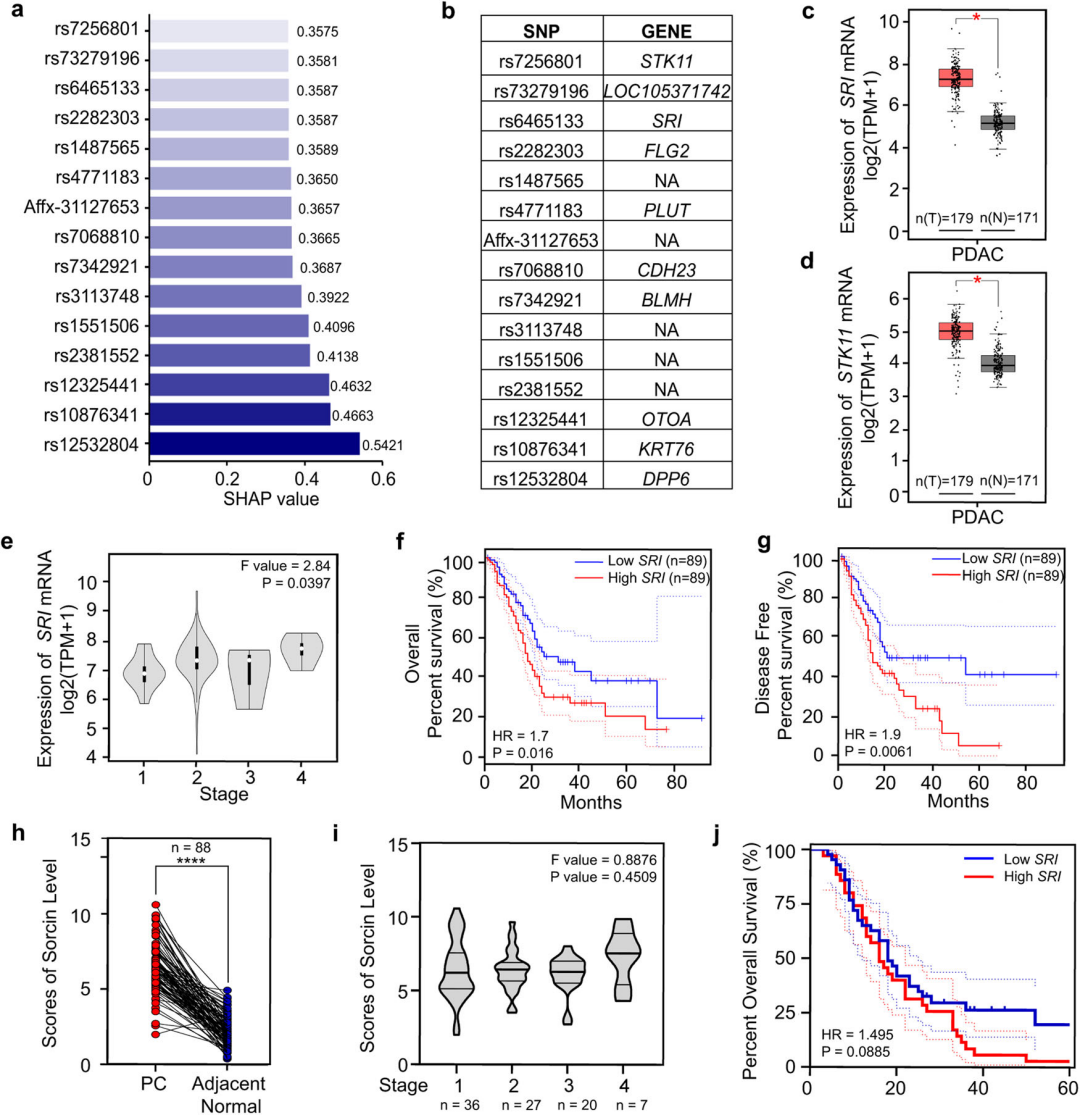
The SNP rs6465133 in *SRI* has the potential to distinguish PCAND from T2DM via machine learning. **a** SHAP values of the 15 SNPs with the greatest significance in distinguishing PCAND from T2DM. **b** The corresponding genes of the top 15 SNPs. The expression patterns of the (**c**) *SRI* and (**d**) *STK11* genes in PC tumors (T) compared with those in normal pancreas tissue (N). **e** The expression pattern of *SRI* in PC patients with different pathological TNM stages. Comparison of (**f**) overall survival rates and (**g**) disease-free survival rates between the high-SRI group and the low-SRI group, with the median SRI expression as the cutoff. Fig. c-g are based on The Cancer Genome Atlas (TCGA) database and Genotype-Tissue Expression (GTEx). **h** Statistical analysis of the sorcin level in pancreatic cancer tissues and adjacent normal tissues from 88 patients diagnosed with PDAC. **i** Statistical analysis of sorcin levels in pancreatic cancer tissues from 88 PDAC patients diagnosed with different pathological TNM stages. **j** Comparison of overall survival rates between the high SRI group (with scores ranging from 712) and the low SRI group (with scores ranging from 06). **h**–**j** are based on PDAC patients enrolled at the Second Affiliated Hospital of Zhejiang University between January 2013 and December 2017. *P < 0.05; ****P < 0.0001, means ± SD are shown. Statistical analysis was performed via Student’s t test for two groups and one-way ANOVA for multiple groups. Kaplan–Meier curves of survival were compared via the log-rank test.

Genomic studies can provide valuable insights into the underlying mechanisms of these phenotypic differences. By leveraging extensive datasets available in large databases (UK Biobank), our objective was to identify difference in the genomic characteristics of PCAND and pure T2DM populations. A total of 12,320 individuals with new-onset diabetes were included in our study, 96 of whom were diagnosed with PCAND and 12,224 of whom were diagnosed with T2DM (Fig. 1a). According to quality control of genetic data and GWAS, a total of 236 SNPs with significant differences between PCAND and T2DM were selected (Fig. 1b). After model construction via machine learning (Fig. 1c), we identified the 15 most meaningful SNPs according to SHAP value (Fig. 2a). Among these 15 SNPs, 10 had corresponding genes (Fig. 2b). We assessed the gene expression patterns of these 10 genes via The Cancer Genome Atlas (TCGA) and Genotype-Tissue Expression (GTEx) databases and found that the expression of only the *SRI* and *STK11* genes was upregulated in PDAC samples compared with normal pancreatic tissues (Fig. 2c-d). There was also a positive correlation between high *SRI* expression and advanced TNM stages of PDAC (F value = 2.84, *P* < 0.005), suggesting a potential role in promoting tumor progression (Fig. 2e). Moreover, elevated expression of *SRI* in PDAC was associated with poor overall survival and disease-free survival (Fig. 2f-g). However, *STK11* expression was not associated with PDAC stage or prognosis. We utilized a dual-luciferase reporter gene system to validate the activity of the SNP rs6465133 as a transcriptional enhancer, as it is present within an intron. The results revealed that, compared with the wild-type plasmid, the mutant plasmid presented greater luciferase activity (Supplementary Fig. 1a-b).

To validate these findings in an independent cohort, we examined biopsies obtained from 88 patients diagnosed with PDAC. Sorcin expression was significantly upregulated in tumor tissues compared with adjacent normal tissues (scores of sorcin levels via IHC, PC tissues vs. paired adjacent normal tissues: 6.49 ± 1.68 vs. 2.18 ± 1.02 (*n* = 88), *P* < 0.0001) (Fig. 2h). However, the expression levels of sorcin were similar in tumor samples of different TNM stages in our cohort (Fig. 2i), probably because the TNM stage is a macroscopic and anatomy-dependent system that may not reflect the cancerous behavior of pancreatic cancer. Otherwise, when the patients were categorized on the basis of sorcin expression in their tumor samples, the high *SRI* group and the low *SRI* group had similar median survival times (16 months in the high *SRI* group vs. 18 months in the low *SRI* group), although the rate of death seemed to decrease for patients in the low *SRI* group once they reached 30 months after surgery (Fig. 2j).

### PC cells inhibit insulin secretion in MIN6 cells in a sorcin-dependent manner in vitro

**Fig. 3:**
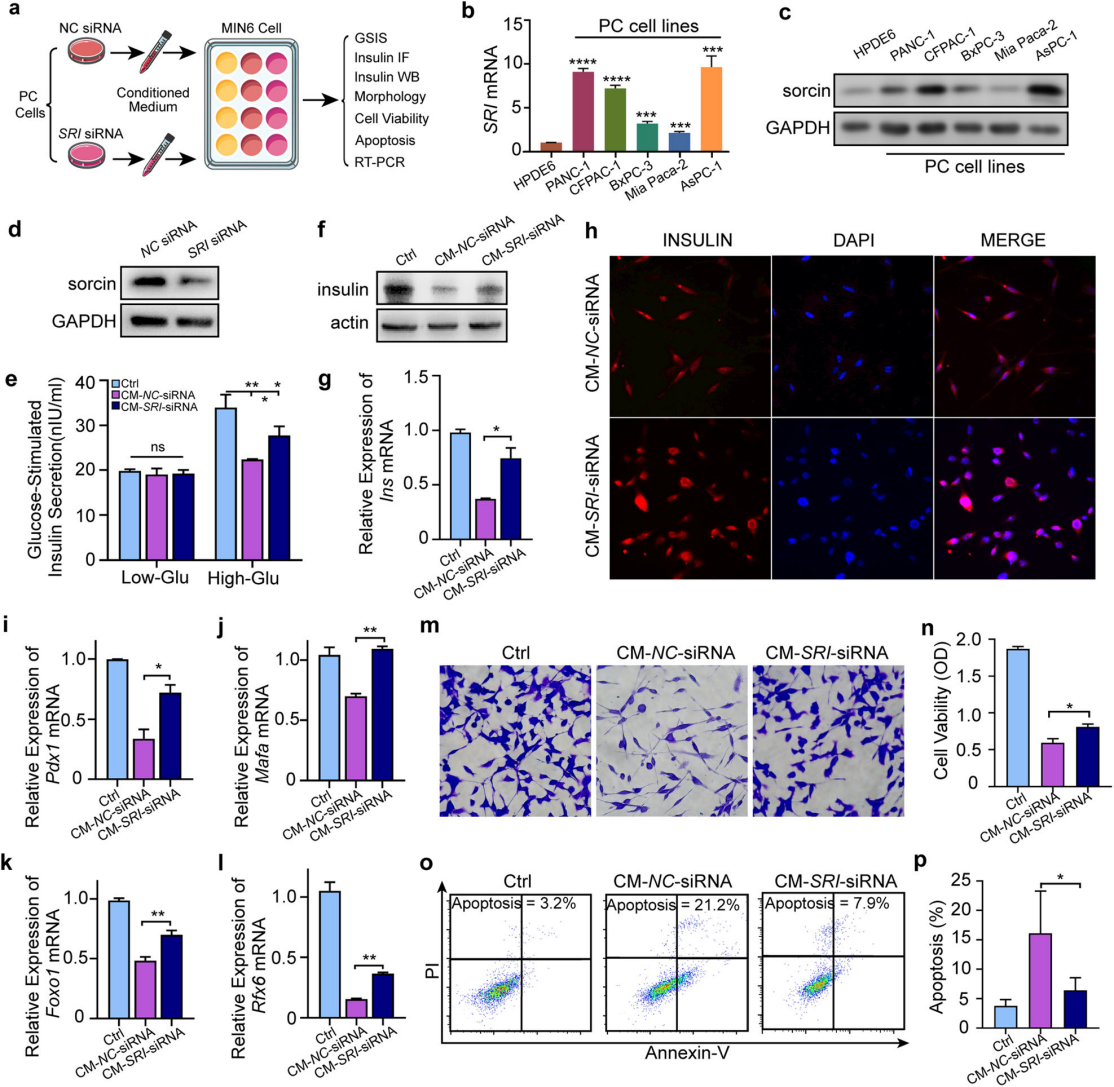
In vitro PC cells inhibit insulin secretion in MIN6 cells in a sorcin-dependent manner. **a** Schematic diagram of the cell experimental design. **b** The mRNA and **c** protein levels of *SRI* in PC cell lines and a normal pancreatic duct cell line (HPDE6). **d** Detection of the knockdown effect in ASPC-1 cells by immunoblotting. **e** Detection of insulin content in the supernatant after GSIS in MIN6 cells incubated with conditioned medium from AsPC-1 cells pretreated with NC siRNA (CM-*NC*-siRNA) or SRI siRNA (CM-*SRI*-siRNA). **f** Detection of insulin content in MIN6 cells incubated with CM-*NC*-siRNA or CM-*SRI*-siRNA from AsPC-1 cells by immunoblotting. **g** Expression of *Ins* mRNA in MIN6 cells incubated with different conditioned media from AsPC-1 cells. **h** Immunofluorescence image showing the content of insulin in MIN6 cells treated with different conditioned media from AsPC-1 cells. The expression of **i** *Pdx1*, **j** *Mafa*, **k** *Foxo1* and **l** *Rfx6* mRNA in MIN6 cells incubated with different conditioned medium from AsPC-1 cells. **m** Morphology of MIN6 cells treated with different conditioned medium from AsPC-1 cells. **n** Assessment of the viability of MIN6 cells treated with different conditioned medium from AsPC-1 cells via MTT assays. **o** Detection of apoptosis via flow cytometry in MIN6 cells treated with different conditioned medium from AsPC-1 cells. **p** Quantification of the percentage of apoptotic cells. Ns, not significant; *P < 0.05; **P < 0.01; ***P < 0.001; ****P < 0.0001, means ± SD are shown. Statistical analysis was performed via Student’s t test for two groups.

Mounting evidence suggests that PCAND is a paraneoplastic phenomenon caused by paracrine factors secreted by cancer or stroma cells^32,33^, some of which have been shown to impinge on β-cells and inhibit insulin secretion^14,20,34^. To investigate how sorcin upregulation may lead to islet dysfunction in PCAND, we utilized in vitro cell cultures to mimic the interactions between pancreatic cancer and islet tissue (Fig. 3a). All five PC cell lines we tested (PANC-1, CFPAC-1, BxPC-3, Mia Paca-2, and AsPC-1) recapitulated the elevated expression of sorcin found in patient tumor samples compared with the normal pancreatic duct cell line HPDE6 (Fig. 3b, c). The same expression patterns were observed in two published external datasets, GSE138437 and GSE166165 (Supplementary Fig. 2a, b). Previous studies have shown that insulin-secreting cell lines, such as MIN6 and INS-1, exhibit impaired glucose-stimulated insulin secretion (GSIS) when cocultured with PC cells or treated with conditioned media from PC cells^14,20,34^. To assess whether high expression of sorcin is required for this process, we performed a knockdown experiment using small-interfering RNAs (siRNAs). Three PC cell lines with particularly high sorcin expression, PANC-1, AsPC-1 and CFPAC-1, were transfected with either siRNA against *SRI* (*SRI*-siRNA) or negative control siRNA (*NC*-siRNA). The knockdown efficiency of *SRI*-siRNA was estimated to be approximately 15%-40% via Western blotting (Fig. 3d and Supplementary Fig. 2c). MIN6 cells exposed to conditioned media collected from *NC*-siRNA-transfected PC cells (CM-*NC*-siRNA) presented a suppressed GSIS response (Fig. 3e and Supplementary Fig. 2d, e), decreased insulin content (Fig. 3f–h and Supplementary Fig. 2f-h) and decreased expression of transcripts related to insulin synthesis and secretion^35–39^ (Fig. 3i–l and Supplementary Fig. 2i–l), as quantified by qRT‒PCR. This phenomenon was accompanied by decreased viability (Fig. 3m, n and Supplementary Fig. 2m, n) and increased apoptosis in MIN6 cells (Fig. 3o, p and Supplementary Fig. 2o, p). The deleterious effects of conditioned media on MIN6 cells were partially rescued when the expression of sorcin in the PC cells was knocked down by *SRI*-siRNA (CM-*SRI*-siRNA in Fig. 3c–o and Supplementary Fig. 2c–p). Together, these results suggest that sorcin is involved in the production of paracrine factors by PC cells, which can negatively impact MIN6 cell viability and the ability of MIN6 cells to synthesize and release insulin.

### PC cells inhibit insulin secretion in pancreatic β-cells in a sorcin-dependent manner in vivo

**Fig. 4:**
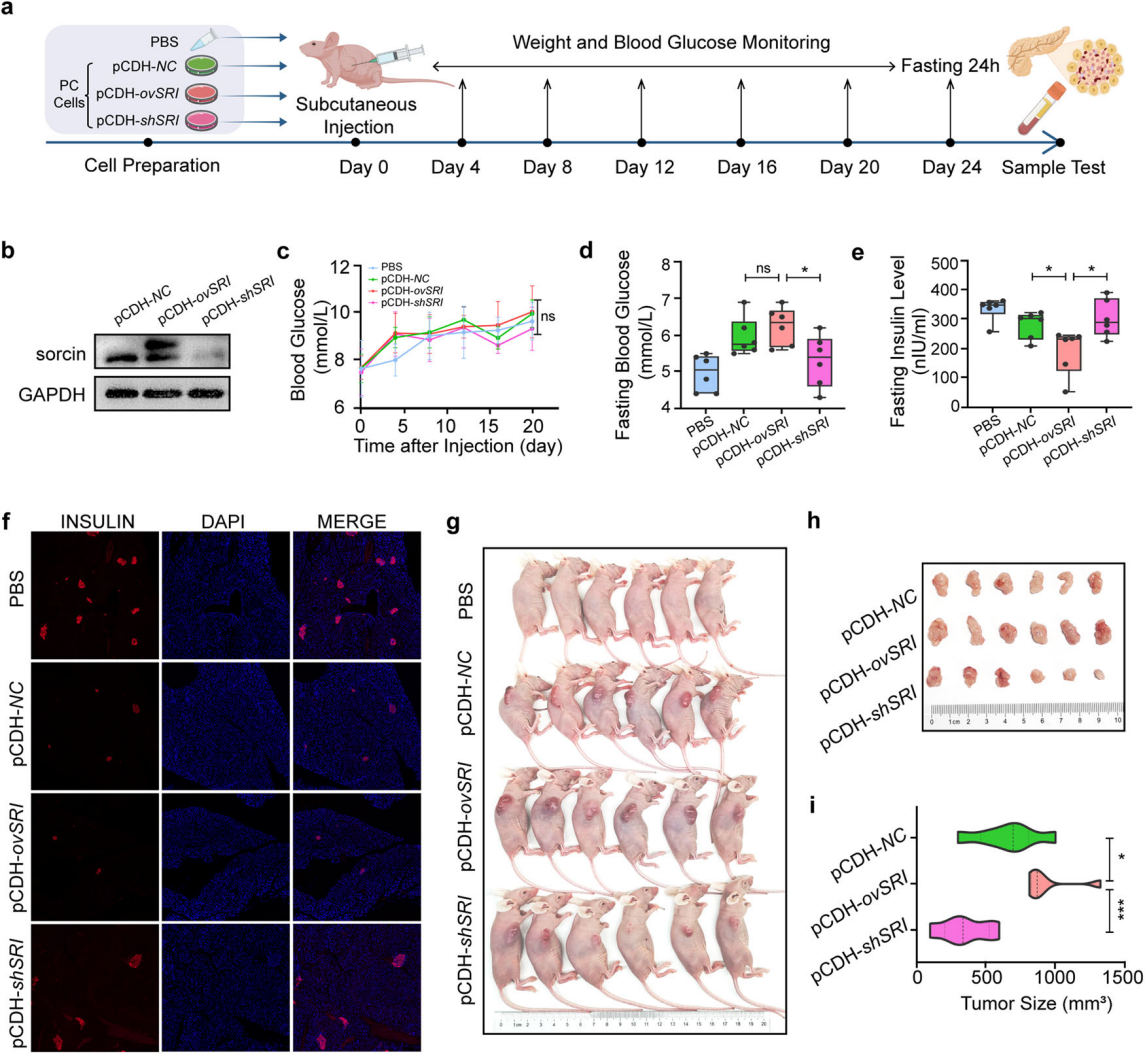
In vivo PC inhibits insulin secretion in pancreatic β-cells in a sorcin-dependent manner. **a** Schematic diagram of the animal experimental design. **b** Detection of the effects of overexpression (pCDH-ov*SRI*) and knockdown (pCDH-*shSRI*) in Pan02 cells by immunoblotting, with pCDH-*NC used* as a control. **c** Blood glucose monitoring was performed every 4 days during subcutaneous tumor formation in nude mice. **d** Fasting blood glucose detection in nude mice after absolute fasting for 24 h before sacrifice. **e** Fasting insulin level detection in nude mice after absolute fasting for 24 h before sacrifice. **f** Immunofluorescence of islets in the pancreas of nude mice labeled with insulin proteins. **g** Photos of subcutaneous tumors in nude mice. **h** Photos of the subcutaneous tumor after surgical removal. **i** Quantitative results of tumor volume. Ns, not significant; *P < 0.05; ***P < 0.001, means ± SD are shown. Statistical analysis was performed via Student’s t test for two groups and one-way ANOVA for multiple groups.

To further elucidate the potential regulatory role of high expression of *SRI* in the damage inflicted by PC cells on islet cells in vivo, we devised a comprehensive research framework (Fig. 4a). We employed the lentivirus transduction technique to generate PC cell lines with either overexpression (pCDH-*ovSRI*) or knockdown (pCDH-*shSRI*) of the *SRI* gene, using an empty plasmid (pCDH-*NC*) as a negative control. The efficiency of overexpression and knockdown was verified by Western blotting (Fig. 4b). Diverging from conventional models employing pancreatic orthotopic tumors associated with PCAND^34^, we opted for a subcutaneous tumor approach to circumvent direct pancreatic injury, and PBS was subcutaneously injected as a blank control. The body weights and blood glucose levels of the nude mice were continuously monitored after subcutaneous injection of pancreatic cancer cells, and the results revealed no significant differences in body weight among the four groups (Supplementary Fig. 3), suggesting the absence of cachexia in the subcutaneous tumor model. However, there were no significant differences in blood glucose levels between the groups (Fig. 4c). This outcome may be attributed to the fact that we did not enforce absolute fasting prior to blood glucose testing. We sacrificed the mice and collected plasma, subcutaneous tumor tissue, and pancreatic tissue when the tumor size was within the tolerable range of that of nude mice and fasted them for 24 h before sacrifice. Interestingly, we found that nude mice bearing pCDH-*ovSRI* tumors had higher fasting blood glucose levels (Fig. 4d) and lower fasting insulin levels (Fig. 4e) than those bearing pCDH-*shSRI* tumors. Moreover, the immunofluorescence results revealed that the islets in the visual field of the pCDH-*ovSRI* group almost disappeared, whereas the islet morphology and insulin signal in the pCDH-*shSRI* group were similar to those in the PBS group (Fig. 4f). The findings from our in vivo experiments indicate that pancreatic islet damage can also occur as a consequence of nonadjacent subcutaneous tumors, suggesting the involvement of blood-mediated processes. However, the precise mediators responsible for this phenomenon require further investigation and exploration. In addition, we observed that the size of the subcutaneous tumors was positively correlated with the *SRI* expression level (Fig. 4g-i), suggesting that *SRI* may also be associated with the proliferation of pancreatic cancer cells.

### Sorcin-overexpressing PC cells release CCL5 and serpin E1 to inhibit insulin secretion in MIN6 cells

**Fig. 5:**
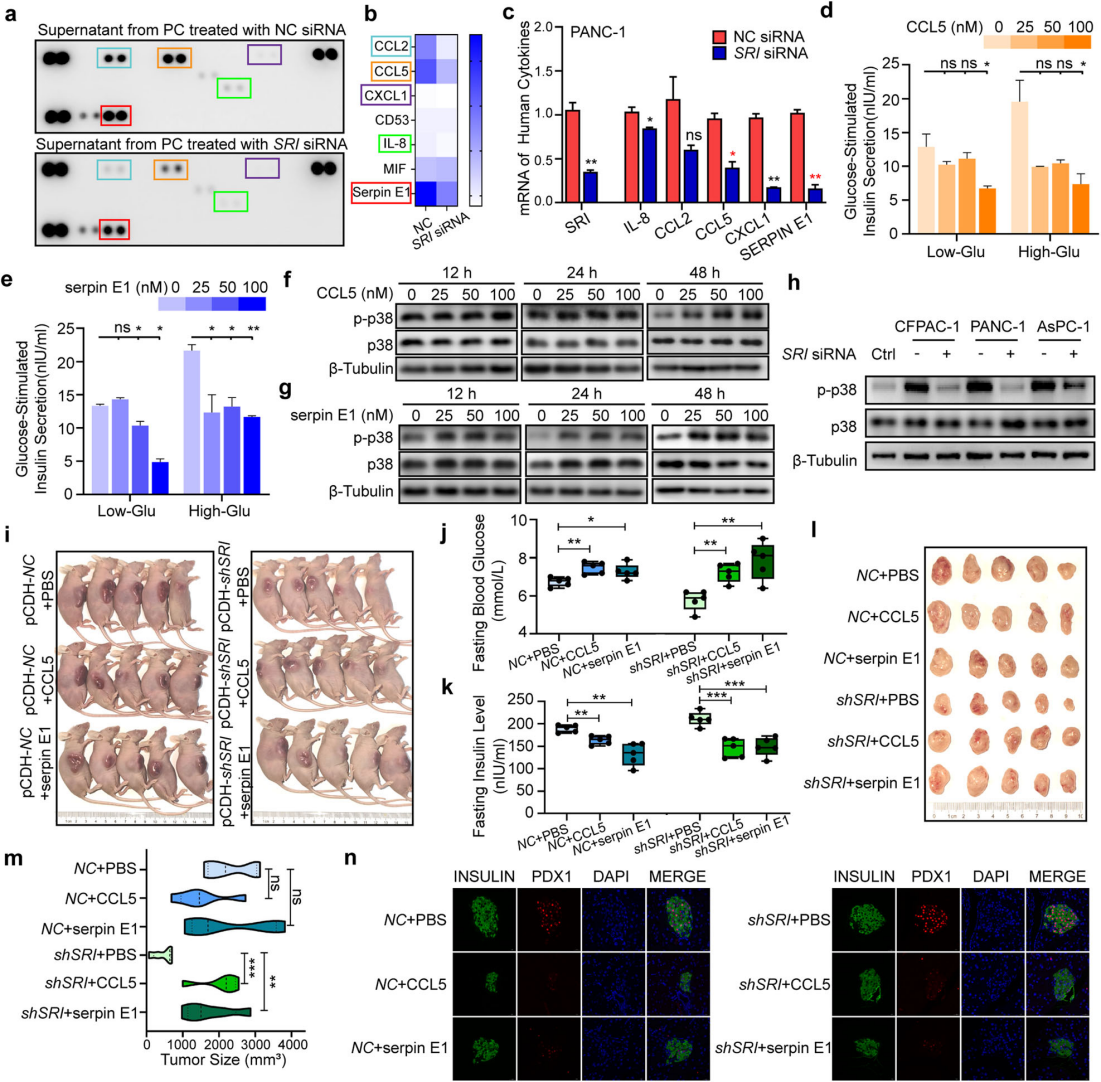
Sorcin-overexpressing PC cells release CCL5 and serpin E1 to inhibit insulin secretion in MIN6 cells. **a** Human cytokine array of supernatants from PANC-1 cells treated with CM-*NC*-siRNA or CM-*SRI*-siRNA. **b** Quantitative analysis of downregulated components in PANC-1 cells treated with *SRI* siRNA in (**a**). **c** Detection of the mRNA levels of *SRI* and 5 downregulated cytokines by RT‒PCR in PANC-1 cells. Fresh culture media containing inflammatory factors was changed every 12 h until 72 h after MIN6 cells were seeded, and the insulin content in the supernatant after GSIS in MIN6 cells was treated with gradient concentrations of (**d**) CCL5 and (**e**) serpin E1. The phosphorylation levels of p38 in MIN6 cells incubated with different concentrations of (**f**) CCL5 and (**g**) serpin E1 at concentrations of 0, 25, 50 and 100 nM. **h** Detection of the phosphorylation level of the p38 pathway in MIN6 cells treated with different conditioned media from PANC-1, CFPAC-1 and AsPC-1 cells for 72 h. **i** Photos of nude mice following subcutaneous injections of pCDH-*NC* or pCDH-*SRI* PC cells and subsequent intratumoral injections of PBS, CCL5, or serpin E1 after tumor formation. **j** Fasting blood glucose detection in nude mice after absolute fasting for 24 h before sacrifice. **k** Fasting insulin level detection in nude mice after absolute fasting for 24 h before sacrifice. **l** Photos of subcutaneous tumors after surgical removal. **m** Quantitative results of tumor volume. **n** Immunofluorescence of islets in the pancreas of nude mice labeled with insulin and PDX1 proteins. Ns, not significant; *P < 0.05; **P < 0.01, means ± SD are shown. Statistical analysis was performed via Student’s t test for two groups.

To further elucidate the mechanism by which conditioned media from PC cells impact β-cells, we performed experiments to identify the paracrine factors released by PC cells under the regulation of sorcin. The pancreatic tumor microenvironment is known to be rich in inflammatory cytokines that support tumor growth^40,41^ and contribute to β-cell dysfunction and apoptosis^42^. To assess the possibility that sorcin-overexpressing PC cells release inflammatory cytokines, we used a human cytokine array to analyze the cytokine profile in the supernatants (conditioned media) collected from PANC-1 cells with and without sorcin knockdown. Five cytokines were significantly downregulated in the *SRI*-siRNA group (Fig. 5a, b). Among them, only *CCL5* and *SERPIN E1* were consistently downregulated in all five PC cell lines following sorcin knockdown (Fig. 5c and Supplementary Fig. 4a–d). Furthermore, we observed a significant increase in the mRNA levels of *CCL5* and *SERPIN E1* after the SRI gene was overexpressed in Mia Paca-2 and BxPC-3 cells (Supplementary Fig. 4e, f). Recombinant CCL5 and serpin E1 proteins inhibited the GSIS response in MIN6 cells in a dose-dependent manner (Fig. 5d, e), confirming the role of these inflammatory cytokines in disrupting β-cell insulin secretion. Moreover, treatment with CCL5 and serpin E1 for prolonged durations ( > 48 h for CCL5 and > 12 h for serpin E1) led to an increase in p38 mitogen-activated protein kinase (MAPK) activation in MIN6 cells (Fig. 5f, g), which has been associated with β-cell apoptosis^43^ and may also underlie the apoptotic phenotype induced by conditioned media from sorcin-overexpressing PC cells (CM-*NC*-siRNA in Figs. 3m–p and 5h). Notably, PC-induced p38 activation in MIN6 cells was attenuated when sorcin expression was knocked down (CM-*SRI*-siRNA in Fig. 5h). Thus, we identified CCL5 and serpin E1 as key components of PC cell secretions that disrupt β-cell functions and identified p38 as a potential downstream target of these inflammatory cytokines in β-cells.

To validate the in vivo effects of the secretion of the inflammatory cytokines downstream of SRI (CCL5 and serpin E1) on islets in situ, we subcutaneously implanted pCDH-*NC* and pCDH-*shSRI* pancreatic cancer cells into nude mice. Following tumor formation, we administered intratumoral injections of CCL5, serpin E1, or PBS (Fig. 5i). Prior to sacrifice, we fasted the mice for 24 h when the tumor size remained within the tolerable range for nude mice and subsequently collected plasma, subcutaneous tumor tissue, and pancreatic tissue. As anticipated, in both sets of nude mice (those harboring pCDH-*NC* and those harboring pCDH-*shSRI* pancreatic cancer cells), intratumoral administration of CCL5 and serpin E1 led to elevated fasting blood glucose levels (Fig. 5j) and reduced fasting insulin levels (Fig. 5k) compared with those in the PBS group. Concurrently, immunofluorescence findings demonstrated that intratumoral injection of CCL5 or serpin E1 diminished insulin signaling within the pancreatic islets in situ and decreased PDX1 signaling, which is linked to insulin synthesis (Fig. 5n). Furthermore, we noted increased sizes of subcutaneous tumors after intratumoral injection of CCL5 or serpin E1, which was particularly evident in the pCDH-shSRI groups (Fig. 5l, m), indicating the potential collaborative effects of SRI and downstream inflammatory cytokines on the proliferation of pancreatic cancer cells.

### Sorcin upregulates CCL5 and serpin E1 expression by forming a positive feedback loop with STAT3

**Fig. 6:**
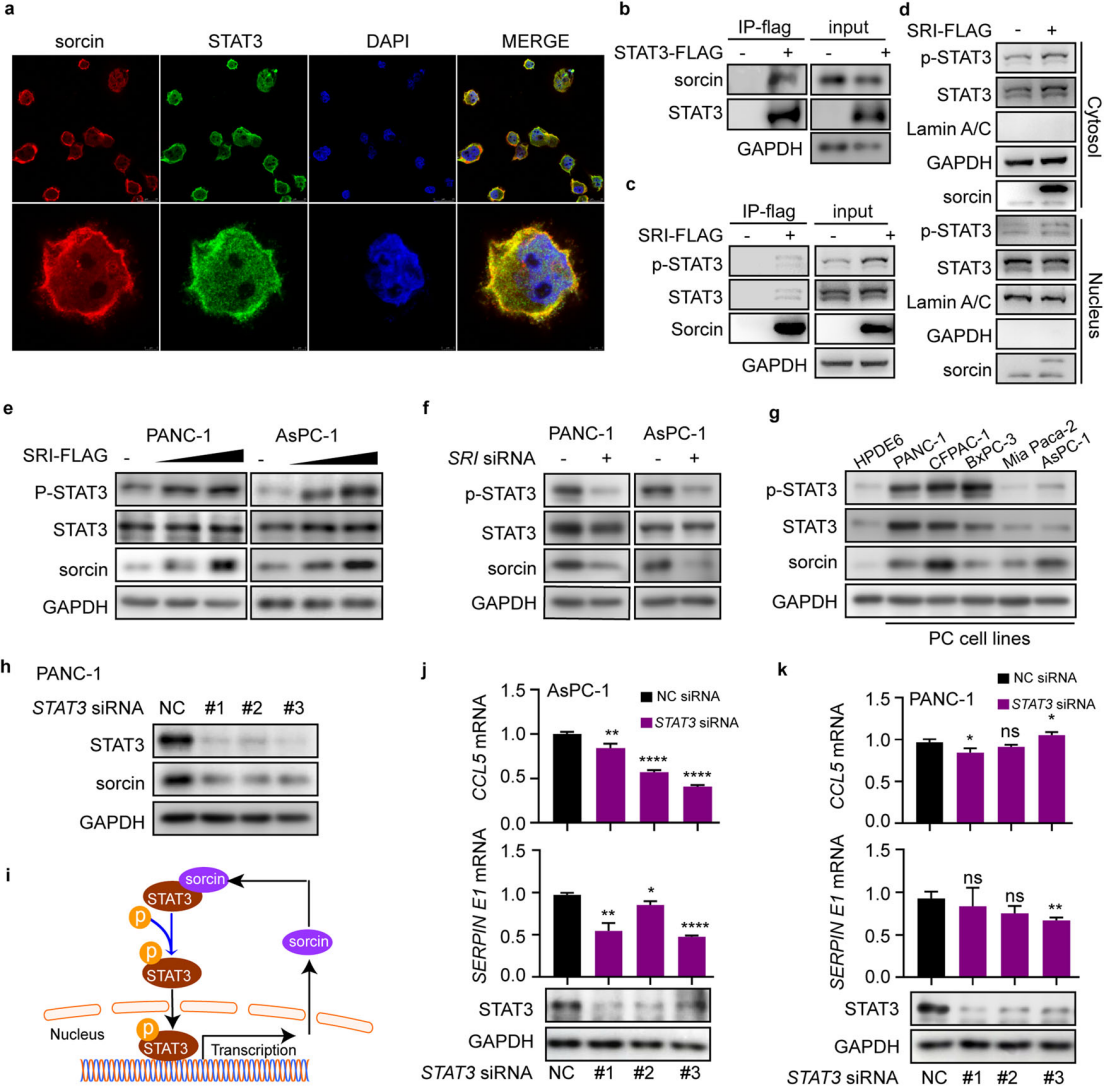
Sorcin upregulates CCL5 and serpin E1 expression by forming a positive feedback loop with STAT3. **a** Confocal immunofluorescence microscopy images of sorcin (red) and STAT3 (green) in PANC-1 cells. **b** Immunoprecipitation of the sorcin-STAT3 complex with anti-flag antibodies after pcDNA-*STAT3*-FLAG plasmid transfection in PANC-1 cells. **c** Immunoprecipitation of the sorcin-STAT3 complex with anti-flag antibodies after pcDNA-*SRI*-FLAG transfection in PANC-1 cells. **d** After PANC-1 cells were transfected with the pcDNA-*SRI*-FLAG plasmid, cytoplasmic separation was performed, and STAT3 and its phosphorylation levels in the nucleus and cytoplasm were detected. **e** Phosphorylation levels of STAT3 in PANC-1 and AsPC-1 cells transfected with gradient increasing pcDNA-*SRI*-FLAG plasmids at different concentrations ranging from 1 to 2 μg/well in 12-well plates; the empty pcDNA vector was used as a control. **f** Phosphorylation levels of STAT3 in PANC-1 and AsPC-1 cells transfected with *SRI* siRNA. **g** The levels of sorcin and STAT3 and the phosphorylation levels of STAT3 in HPDE6 cells and 5 kinds of PC cells. **h** The level of sorcin in PANC-1 cells transfected with *STAT3* siRNA. **i** A schematic diagram of the sorcin-STAT3 positive feedback loop. Detection of *CCL5* and *SERPIN E1* mRNA and the expression levels of STAT3 after treatment with *STAT3* siRNA in (**j**) AsPC-1 and (**k**) PANC-1 cells. Ns, not significant; *P < 0.05; **P < 0.01; ***P < 0.001; ****P < 0.0001, means ± SD are shown. Statistical analysis was performed via Student’s t test for two groups.

Thus far, we have shown that the overexpression of sorcin in PC cells leads to increased secretion of CCL5 and serpin E1, which act on nearby β-cells. Since sorcin itself is not known to be a transcription factor, we speculated that it may interact with one or more transcription factors to upregulate CCL5 and serpin E1 expression in PC cells. Indeed, sorcin has been reported to interact with signal transducer and activator of transcription 3 (STAT3) in mouse hepatocytes^44^. In PC cells, sorcin and STAT3 colocalize (Fig. 6a) and can be coimmunoprecipitated as a protein complex (Fig. 6b, c). Specifically, following transfection with the pcDNA-*SRI*-FLAG plasmid, immunoprecipitation analysis revealed the presence of STAT3, along with its phosphorylated form, in complex with sorcin protein (Fig. 6c). The phosphorylation level of STAT3 appeared to be dictated by the expression level of sorcin, and the level of phospho-STAT3 (p-STAT3) was increased after sorcin overexpression in both the nucleus and cytoplasm (Fig. 6d). In PANC-1 and AsPC-1 cells, the p-STAT3 level increased with pcDNA-*SRI*-FLAG transfection in a concentration-dependent manner (Fig. 6e) and decreased with siRNA-mediated sorcin knockdown (Fig. 6f). Similarly, PC cell lines with elevated sorcin expression (Fig. 3b-c) presented higher levels of p-STAT3 than did the normal pancreatic duct epithelial cell line HPDE6 (Fig. 6g). In PDAC tumor tissues from human patients, sorcin was highly expressed in the cytoplasm of PC cells, while p-STAT3, an activated transcription factor, was enriched in the nucleus (Supplementary Fig. 5a). Interestingly, when STAT3 expression was knocked down by siRNAs in PANC-1 cells, sorcin expression was also largely diminished (Fig. 6h), suggesting that STAT3, in turn, increased the expression level of sorcin. Thus, the synergistic interactions between sorcin and STAT3 form a positive feedback loop (Fig. 6i), resulting in the sustained overexpression of sorcin and the activation of STAT3 in PC cells.

To further confirm that the sorcin-STAT3 loop is responsible for increasing the transcription of CCL5 and serpin E1 in PC cells, we examined the effect of STAT3 knockdown on *CCL5* and *SERPIN E1* transcript levels. In AsPC-1 and CFPAC-1 cells, STAT3 knockdown via three different siRNAs resulted in the downregulation of *CCL5* and *SERPIN E1* transcripts (Fig. 6j, k and Supplementary Fig. 5b), similar to what we observed with sorcin knockdown (Fig. 5c and Supplementary Fig. 4a–d). In PANC-1 cells, on the other hand, the impact of STAT3 knockdown on *CCL5* mRNA levels varied with different siRNAs, while all three *STAT3*-siRNAs led to a slight but significant decrease in *SERPIN E1* mRNA. This discrepancy is perhaps not surprising, considering that STAT3 knockdown not only affects gene targets directly downstream of the sorcin-STAT3 loop (Fig. 6k) but also disrupts the interactions between STAT3 and other proteins^45^.

### In the clinical cohort, *SRI* differentiated between PCAND and T2DM, and downstream serpin E1 may be a potential biomarker

**Fig. 7:**
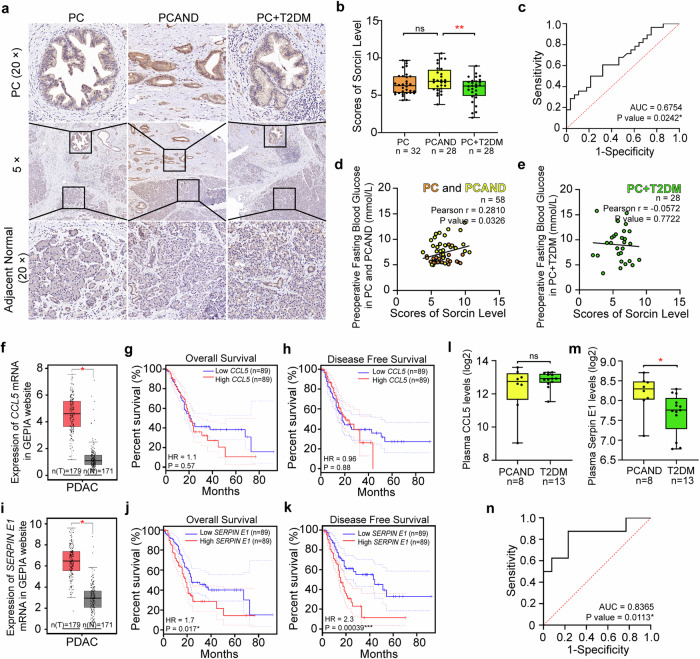
In the clinical cohort, *SRI* can be used to differentiate PCAND from T2DM, and downstream serpin E1 may be a potential biomarker. **a** Representative images of sorcin staining in tumor tissues and adjacent normal tissues from patients with pure pancreatic cancer (PC), pancreatic cancer with new-onset diabetes (PCAND) and pancreatic cancer with long-term diabetes (PC + T2DM). **b** Statistical analysis of sorcin levels in pancreatic cancer tissues from patients with PC, PCAND and PC + T2DM. **c** The sensitivity and specificity in discriminating PCAND patients from the PC + T2DM population for sorcin are shown. The ROC curves were generated and AUCs were calculated for 28 PCADN patients and 28 PC + T2DM patients. Correlation analysis of fasting glucose levels before pancreatic tumor resection and sorcin immunohistochemical scores in (**d**) PDAC patients without T2DM (pure PC and PCAND, green points) and **(e)** with T2DM (PC + T2DM, orange points). **f** The expression of *CCL5* mRNA in PC tumors (T) compared with that in normal pancreatic tissue (N). Comparison of (**g**) overall survival rates and (**h**) disease-free survival rates between the high *CCL5* group and the low *CCL5* group, with the median *CCL5* expression level used as the cutoff. **i** The expression of *SERPIN E1* mRNA in PC tumor tissue (T) compared with normal pancreas tissue (N). Comparison of (**j**) overall survival rates and (**k**) disease-free survival rates between the high-*SERPIN E1* group and the low-*SERPIN E1* group, with the median *SERPIN E1* expression level used as the cutoff. F-K is based on The Cancer Genome Atlas (TCGA) database and the Genotype-Tissue Expression (GTEx) dataset from the GEPIA website. The plasma (**l**) CCL5 and (**m**) serpin E1 levels in patients with pancreatic cancer-associated new-onset diabetes (PCAND) and type 2 diabetes (T2DM). **n** The sensitivity and specificity in discriminating PCAND patients from the DM population for serpin E1 are shown. The ROC curves were generated and AUCs were calculated for 8 PCADN patients and 13 pure T2DM patients. Ns, not significant; *P < 0.05; **P < 0.01; ***P < 0.001, means ± SD are shown. Statistical analysis was performed via Student’s t test for two groups. Kaplan–Meier curves of survival were compared via the log-rank test.

In our previous cohort of 88 patients with PDAC, patients were further classified into three groups on the basis of their diabetes status: those with no diabetes (pure PC), those with PCAND, and those with long-term diabetes (PC + T2DM). Notably, a greater level of sorcin expression was detected in PCAND tumor tissues than in PC + T2DM tumor tissues (sorcin IHC scores, PCAND vs. PC + T2DM: 7.10 ± 1.71 (*n* = 28) vs. 5.85 ± 1.67 (*n* = 28), *P* = 0.008; pure PC vs. PCAND: 6.51 ± 1.51 (*n* = 32) vs. 7.10 ± 1.71 (*n* = 28), *P* = 0.136) (Fig. 7a, b). The area under the curve (AUC) for sorcin in differentiating between PCAND patients and PC + T2DM patients was 0.675 (*P* = 0.02423, 95% CI 0.5358-0.8150) (Fig. 7c). Furthermore, fasting blood glucose levels in patients with pure PC and PCAND before pancreatectomy were positively correlated with sorcin expression levels (Pearson correlation coefficient between sorcin IHC scores and fasting blood glucose level *r* = 0.281, *P* = 0.0326) (Fig. 7d), which was not observed in PC + T2DM patients (Pearson correlation coefficient between sorcin IHC scores and fasting blood glucose level, *r* = -0.0572, *P* = 0.7722) (Fig. 7e), suggesting a potential link between the upregulation of sorcin and islet dysfunction specific to PCAND. Interestingly, in rare instances in which the pancreatic tissue section included both the PDAC tumor and the adjacent islets, high sorcin levels in PDAC tumors coincided with low insulin levels and PDX1 levels in tumor-adjacent islets (Supplementary Fig. 6a, b). However, since this phenomenon was observed in only two available sections, the conclusion may not be solid and only partially suggests that decreased insulin secretion is likely responsible for the increased fasting blood glucose level in patients with high sorcin expression.

According to the TCGA and GTEx data, both *CCL5* and *SERPIN E1* were expressed at significantly higher levels in pancreatic cancer tissues (T, *n* = 179) than in nearby normal pancreatic tissues (N, *n* = 171) (Fig. 7f, i). Among PDAC patients, high *SERPIN E1* expression was associated with a poor prognosis (Fig. 7j, k). The differences between the high *CCL5* and low *CCL5* groups was not statistically significant, although the overall and disease-free survival rates did appear to be greater for patients in the low *CCL5* group after 20 months (Fig. 7g, h). To assess the performance of CCL5 and serpin E1 as potential biomarkers for PCAND, we measured their concentrations in the peripheral blood of patients diagnosed with either PCAND (*n* = 8) or T2DM (*n* = 13). There was no difference in CCL5 expression between the two groups (Fig. 7l). This may be related to the fact that another important source of CCL5 is adipose tissue, which has a relatively high concentration in T2DM patients, resulting in its insufficient potential as a biomarker of PCAND^46,47^. However, the level of serpin E1 was significantly greater in PCAND than in pure T2DM (Fig. 7m). In this small cohort, serpin E1 achieved an AUROC of 0.8364 in differentiating between PCAND and pure T2DM (*P* = 0.0113, 95% CI 0.6415–1.000) (Fig. 7n), demonstrating its potential utility as a biomarker for PCAND.

## Discussion

Pancreatic cancer (PC), a devastating disease characterized by late diagnosis, limited treatment success and a dismal prognosis, remains a major medical challenge. A rise in blood glucose is one of the early warning signs of underlying PC and may be an indicator of genetic events in PC progression. Considering the convenience and popularity of blood glucose monitoring, one of the keys to early diagnosis of PC is to identify the small subset (1%) of PCAND patients among the new-onset diabetes population as early as possible^48–50^. An improved understanding of the molecular mechanisms and signaling pathways underlying its specific pathogenesis is needed to support progress in PCAND detection.

The SHAP technique is a method used to interpret the optimal model output, and it has been used to select important features for clinical prediction models in some studies^51,52^. A recent study used machine learning and SHAP technology to predict patients with new-onset diabetes at risk of PC^53^. However, this study focused only on clinical indicators and did not consider genomic factors. In this study, we employed machine learning techniques to identify SNPs and their corresponding genes that may be used to distinguish PCAND from T2DM. We further mapped a novel sorcin-STAT3-serpin E1/CCL5 signaling axis in PC cells, which explains how early presymptomatic PC may coincide with new-onset diabetes in some patients^54^. Sorcin and STAT3 form a positive feedback loop to increase the transcription of serpin E1 and CCL5. These inflammatory cytokines released by PC cells can impair nearby islet β-cells, likely by activating the p38 signaling pathway. In addition, in biopsies obtained from 88 PDAC patients, we detected elevated expression of sorcin in pancreatic cancer tissues, especially in PCAND. These results suggest that sorcin may be the key driver in PCAND and that aberrant activation of the sorcin-STAT3-serpin E1/CCL5 signaling axis likely underlies PCAND pathogenesis.

**Fig. 8:**
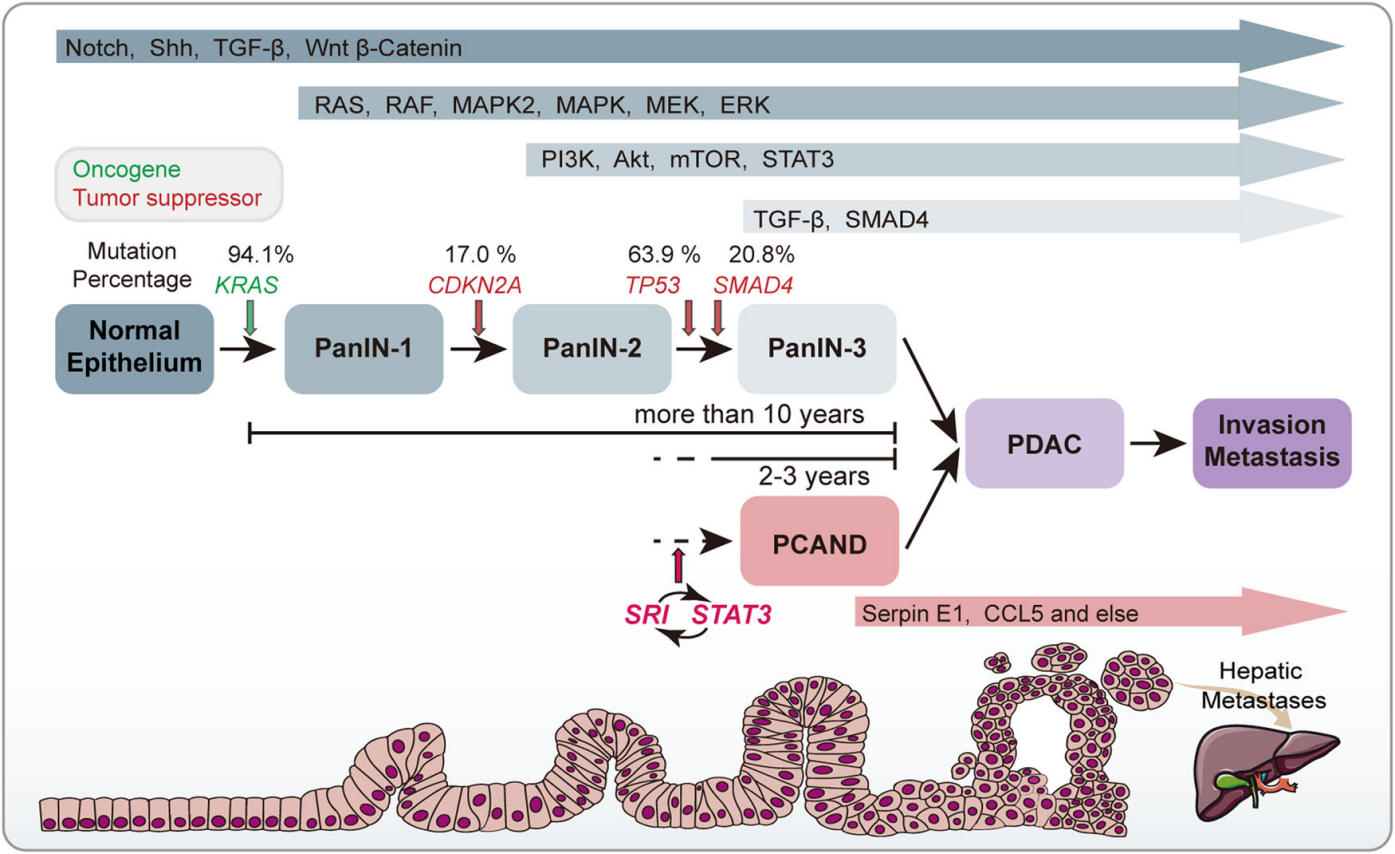
*SRI* may be the driver gene in the pathogenesis of PCAND. During PC genesis and development, a variety of driver gene mutations (*KRAS*, *CDKN2A*, *TP53* and *SMAD4*) and signaling pathway deregulation participate in the progression from pancreatic intraepithelial neoplastic lesions (PanINs 1-3) to PDAC. *KRAS* mutation is the initiating genetic event for PC, and the progression of normal pancreatic tissue to PDAC involves a stepwise genetic transition projected to span more than 10 years. PCAND is defined as PDAC with new-onset diabetes diagnosed within 2–3 years prior, so abnormal activation of the sorcin-STAT3 positive feedback loop may occur during the progression from PanIN 1-3 to PDAC, later than oncogene mutation. Downstream inflammatory cytokines/chemokines (such as CCL5 and serpin E1) may be potential biomarkers.

While exploring the driver mechanism of *SRI* in PCAND, we identified potential bidirectional crosstalk between PCAND pathogenesis and inflammation, which is likely regulated by the sorcin-STAT3-serpin E1/CCL5 signaling axis. Interestingly, the signaling axis we describe here shares a common critical node, STAT3, with the inflammatory pathway downstream of KRAS, whose mutations are the most common genetic abnormality in PC^6^. Given the long duration (over 10 years for PC development)^55^ and lack of specificity of *KRAS* mutation in the detection of PC progression^8^, screening or diagnostic use of these mutations in the clinic is limited. In this study, we found that fasting blood glucose levels in pure PC and PCAND patients before pancreatectomy were positively correlated with sorcin expression levels. Therefore, the increase in blood glucose driven by *SRI* gene could be due to the externalization of PCAND, which typically manifests 2–3 years prior to the diagnosis of PC^25^, most likely during the progression from PanIN-3 to PC (Fig. 8)^5,56–61^. These results further support the notion that early screening strategies based on *SRI* gene may be better than those based on *KRAS* and other oncogenes that are mutated in the early PanIN stage.

Previous research has suggested that sorcin acts as a protective factor in β-cells in T2DM^62^. However, our in vitro research revealed that PC-derived sorcin plays a negative role in β-cell function and can induce inflammatory damage. Unlike T2DM with adipocyte-derived inflammatory cytokines^63^, PC has a specific inflammatory tumor microenvironment (TME)^64^. This study indicated that the increased secretion of serpin E1 and CCL5 induced by the sorcin-STAT3 interaction may in turn contribute to the formation of an inflammatory TME^65^, alongside *KRAS*-associated inflammatory signaling^66^. Furthermore, on the basis of large-scale cohorts from the UK Biobank, we confirmed that the *SRI*-based model is superior to models based on other driver genes, such as *KRAS*, in differentiating PCAND from T2DM and that the combination of *SRI*, *KRAS*, and *CDKN2A* with a clinical model can further increase the efficiency. On the other hand, on the basis of a small cohort of PCAND and T2DM patients, the concentration of serpin E1 in peripheral blood samples showed decent diagnostic performance. We are aware that this is a preliminary study that has several limitations, such as the sample size of the clinical cohorts. As a next step, we believe that a larger-scale validation study with a longitudinal sampling scheme should be carried out in the future.

In summary, GWAS analysis and machine learning based on a large-scale database identified a SNP (rs6465133) in the *SRI* gene whose frequency was significantly different between the PCAND and T2DM populations. Further biological experiments revealed a novel sorcin-STAT3-serpin E1/CCL5 signaling axis as a key driver of PCAND pathogenesis. The convergence of sorcin and KRAS signaling on STAT3 suggests potential bidirectional crosstalk, which should be considered when selecting targeted therapies for PC involving these pathways. Our results also suggest that plasma serpin E1 may be a potential biomarker for PCAND. Further studies on the molecules downstream of the sorcin pathway may yield valuable clues for the early diagnosis of PC.

## Supplementary information

Supplementary Information

## Data Availability

All data are available in the main text or the supplementary materials.
