# Author Correction: Sorcin can trigger pancreatic cancer-associated new-onset diabetes through the secretion of inflammatory cytokines such as serpin E1 and CCL5

**DOI:** 10.1038/s12276-024-01363-3

**Published:** 2024-11-28

**Authors:** Jiali Gong, Xiawei Li, Zengyu Feng, Jianyao Lou, Kaiyue Pu, Yongji Sun, Sien Hu, Yizhao Zhou, Tianyu Song, Meihua Shangguan, Kai Zhang, Wenjie Lu, Xin Dong, Jian Wu, Hong Zhu, Qiaojun He, Hongxia Xu, Yulian Wu

**Affiliations:** 1 Second Affiliated Hospital, Zhejiang University School of Medicine, Hangzhou, Zhejiang China; 2 Key Laboratory of Cancer Prevention and Intervention, China National Ministry of Education, Cancer Institute, Second Affiliated Hospital, Zhejiang University School of Medicine, Hangzhou, Zhejiang China; 3 Cancer Center, Zhejiang University, Hangzhou, Zhejiang China; 4 Department of Surgery, Fourth Affiliated Hospital, Zhejiang University School of Medicine, Yiwu, Zhejiang China; 5 Harvard T.H. Chan School of Public Health, Harvard University, Boston, MA USA; 6 School of Public Health and Eye Center The Second Affiliated Hospital, Zhejiang University, Hangzhou, China; 7 School of Public Health, Zhejiang University School of Medicine, Hangzhou, Zhejiang China; 8 Institute of Wenzhou, Zhejiang University, Wenzhou, Zhejiang China; 9 Zhejiang Province Key Laboratory of Anti-Cancer Drug Research, College of Pharmaceutical Sciences, Zhejiang University, Hangzhou, Zhejiang China; 10 Center for Drug Safety Evaluation and Research of Zhejiang University, Hangzhou, Zhejiang China; 11 Innovation Institute for Artificial Intelligence in Medicine and Liangzhu Laboratory, Zhejiang University School of Medicine, Zhejiang University, Hangzhou, China

**Keywords:** Diagnostic markers, Cancer genetics, Tumour biomarkers

Correction to: *Experimental & Molecular Medicine* 10.1038/s12276-024-01346-4, published online 08 November 2024

In this article, Jiali Gong, Xiawei Li, Zengyu Feng, and Jianyao Lou should have been denoted as equally contributing authors. The original article has been corrected.

