# Supplementary material for: Sorcin can trigger pancreatic cancer-associated new-onset diabetes through the secretion of inflammatory cytokines such as serpin E1 and CCL5
Source: Exp Mol Med. 2024 Nov 8;56(11):2535–47. doi: 10.1038/s12276-024-01346-4 (PMC11612510; doi:10.1038/s12276-024-01346-4)
Supplement: Supplementary file 1 — Supplementary Information [file 12276_2024_1346_MOESM1_ESM.pdf]

SUPPLEMENTARY FILES

Supplementary Materials and Methods

Plasmid and small interfering RNA (siRNA) transfection

Human pancreatic cancer cells were transfected with plasmids using JetPRIME transfection reagent (Polyplus, Beijing, China). Human pancreatic cancer cells were transfected with siRNA using INTERFERin transfection reagent (Polyplus, Beijing, China) according to the manufacturer's instructions. The pcDNA-*SRI*-FLAG plasmid was purchased from WZ Biosciences Inc. (Shangdong, China) and pcDNA-*STAT3*-FLAG plasmid was kindly provided by Prof. Hong Zhu (Zhejiang University, Hangzhou, China). The sense strands of the duplex siRNAs (synthesized by GenePharma, Shanghai, China) were: *NC:* 5'-UUCUCCGAACGUGUCACGUTT-3', *SRI*: 5'-GCCGGCUUAUGGUUUCAAUTT-3', *STAT3* #1: 5'-GGGACCUGGUGUGAAUUAUTT-3', *STAT3* #2: 5'-CCCGGAAAUUUAACAUUCUTT-3', *STAT3* #3: 5'-GGUACAUCAUGGGCUUUAUTT-3'.

Dual-luciferase reporter gene experiment

The firefly luciferase reporter gene was amplified by polymerase chain reaction (PCR) from genomic DNA and cloned into the multiple cloning site of the pGL3-promoter vector. The resulting plasmid was verified by sequencing (Supplementary Fig. 1a). For transfection, HEK293T cells were seeded in 24-well plates and allowed to reach 70-80% confluency. The cells were then transfected with 500 ng of the plasmid above using JetPRIME transfection reagent (Polyplus, Beijing, China) according to the manufacturer's instructions. After 48 hours post-transfection, the cells were lysed, and the lysates were assayed for firefly and

Renilla luciferase activities using tDual-Lumi™ II Luciferase Reporter Gene Assay Kit (Beyotime, Shanghai, China) according to the manufacturer's instructions. Firefly luciferase activity was measured first by adding the substrate, followed by quenching and measuring Renilla luciferase activity. The relative luciferase activity was calculated as the ratio of firefly to Renilla luciferase activity.

Collection of conditioned media

The culture media of transfected PC cells were changed to RPMI-1640 without FBS at 6 hours after transfection. After 2 days of culturing, the media were collected and centrifuged at 3000 rpm for 15 min at 4°C. The supernatants (conditioned media) were stored at −80 °C and used within one month. In all subsequent experiments, MIN6 cells were incubated with conditioned media supplemented with 5% FBS.

MTT assay for cell viability

MIN6 cells were seeded in 96-well plates (5000 cells/well) and incubated in different conditioned media for 72 hours, with medium change every 24 hours. Then 20 μL of MTT stock solution was added to the culture medium. After incubating at 37 °C for 4 hours, the medium was completely removed, and 200 μL of DMSO were added to the cells to dissolve the violet crystals. Optical density (OD) values were measured using a microplate reader.

Apoptosis Detection

MIN6 cells were seeded in 12-well plates (1×10^5 cells/well) and incubated in different conditioned media for 72 hours, with medium change every 24 hours. The cells were then harvested by trypsin digestion. Apoptotic cells were stained using the Annexin V-PI apoptosis detection kit (Yeasen, Shanghai, China) and immediately analyzed by flow cytometry.

Co-immunoprecipitation

Cells were lysed with IP lysis buffer (Beyotime Biotechnology, Shanghai, China) supplemented with a protease inhibitor mixture (Thermo, USA) on ice for 30 minutes and then centrifuged at 14,000 g for 10 minutes at 4°C to pellet cell debris. Add anti-FLAG sepharose beads (Cell Signaling Technology, USA) and incubate with rotation for 12 hours at 4°C and then centrifuge at 2,500 g for 5 minutes at 4°C to pellet the beads. Wash beads 3 times with IP lysis buffer. Add elution buffer and incubate for 5 minutes. Finally, subject the eluate to SDS-PAGE and Western blotting to detect co-immunoprecipitated.

Western blot analysis

Cells were lysed with RIPA lysis buffer (Sigma-Aldrich, USA) supplemented with a protease inhibitor mixture (Thermo, USA), and proteins were quantified using the BCA assay. The experimental procedures were performed in accordance with standard protocols. Briefly, proteins were separated on 15% SDS-polyacrylamide gels and blotted onto polyvinylidene difluoride (PVDF) membranes. Membranes were blocked with 5% milk, incubated with specific primary antibodies, horseradish peroxidase (HRP)-conjugated secondary antibodies, and subsequently subjected to chemiluminescence detection. The antibodies used and the corresponding dilutions are listed in Supplementary Table 1.

Quantitative real-time PCR (qRT-PCR)

Gene expression levels were assessed using qRT-PCR. Total RNA was extracted from PC cells and MIN6 cells with TRIzol reagent (Invitrogen, USA). The cDNA templates were synthesized with PrimeScript RT Reagent Kit (TaKaRa, China), and qRT-PCR was performed with a 7500 Fast™ System (Applied Biosystems, USA). *GAPDH* was chosen as an

endogenous control. Data were analyzed using the $2^{-\Delta\Delta Ct}$ method. Specific miRNA primers are listed in Supplementary Table 2.

Immunofluorescence and confocal microscopy

Immunofluorescence analysis was performed to measure insulin levels in MIN6 cells. After 72 h of culturing with different conditioned medium, the cells were washed and fixed in prechilled methanol for 10 min and then permeabilized with 0.1% Triton X-100 for another 10 min. After blocking with 5% BSA, the cells were incubated overnight at 4 °C with an anti-insulin Ab. The primary Ab was detected by cy3-conjugated goat anti-rabbit IgG goat polyclonal antibody for 1 h (HUABIO, Hangzhou). Cell nuclei were stained with DAPI. Observation and image acquisition were performed using a confocal microscope (Zeiss, Germany).

Immunofluorescence analysis was also performed to measure insulin, pdx1 sorcin levels in pathological section of clinical pancreatic cancer and pancreas sample section of nude mouse. Operation method is the same as above. The antibodies used and the corresponding dilutions are listed in Supplementary Table 1.

Immunohistochemistry and scoring standards

Immunohistochemical analysis was performed to assess sorcin, p-STAT3 and insulin expression levels in these samples. Paraffin-embedded tissues were cut into 4 μm sections and deparaffinized. The sections were incubated with anti-sorcin, anti-p-STAT3 and anti-insulin antibodies respectively overnight at 4 °C. The antibodies used and the corresponding dilutions are listed in Supplementary Table 1. After incubation with biotinylated goat anti-mouse IgG for 30 min at 37 °C, each slide was rinsed in phosphate-buffered saline and incubated in the

streptavidin-biotinylated horseradish peroxidase complex for 30 min at 37 °C. The slides were developed using diaminobenzidine substrate solution to visualize peroxidase signals and then counterstained with hematoxylin. These immunohistochemical stains were evaluated by three experienced pathologists independently in a blinded manner, and graded on a semiquantitative scale according to the percentage (P) of stained area (0% area stained = 0, 1–25% area stained = 1, 26–50% area stained = 2, and 51–100% area stained = 3) and the intensity (I) of staining (no staining = 0, weak staining = 1, moderate staining = 2, and strong staining = 3). Results were scored by multiplying the percentage of stained area by the intensity (P × I; maximum = 12). All cases were divided into two groups: a high expression group (with a score ranging from 7 to 12) and a low expression group (with a score ranging from 0 to 6).

Glucose-stimulated insulin release assay (GSIS)

To set up the GSIS assay, MIN6 cells were seeded in 12-well plates (1×10^5 cells/well) and incubated in different conditioned media for 72 hours, with media change every 24 hours. For the GSIS assay, the cells were pre-incubated in Krebs-Ringer bicarbonate buffer (KRBB: 119 mM NaCl, 2.5 mM $CaCl_2$, 1.19 mM KH_2PO_4, 1.19 mM Mg_2SO_4, 10 mM HEPES [pH 7.4], and 1% bovine serum albumin) for 1 hour at 37 °C, then switched to KRBB containing 5.6 mM of glucose (“low-Glu”) for 1 hour and then 16.7 mM of glucose (“high-Glu”) for another hour. The buffers were collected separately, and centrifuged at 1500 rpm for 15 min. All supernatants were stored at −80 °C and used within 2 weeks. The amount of insulin released into the KRBB buffer were determined using an ELISA kit (CUSABIO, Wuhan).

Lentiviral vector and short hairpin RNA (shRNA) transfection

The *SRI*-overexpression plasmid was purchased from WZ Biosciences Inc. (Shangdong, China). We constructed short hairpin RNA (shRNA) expression vectors to knock down *SRI* expression. The shRNA primer sequences for *SRI* are 5’-CCGGGATCCGCTGTATGGTTACTTTCTCGAGAAAGTAACCATACAGCGGATCTTTTTG-3’ (Forward) and5’-AATTCAAAAAGATCCGCTGTATGGTTACTTTCTCGAGAAAGTAACCATACAGCGGATC-3’ (Reverse). The lentiviral vector was co-transfected with packaging vectors into 293T cells. The PANC-02 cells were infected with empty, *SRI*-overexpression and *SRI*-knockdown lentiviruses. The PANC-02 cells with a stable overexpression of *SRI* were termed as pCDH-*ovSRI*, and the PANC-02 cells with a stable konckdown of *SRI* were termed as pCDH-*shSRI*, and the PANC-02 cells transfected with the empty vector were referred to pCDH-*NC*.

Supplementary Tables

Supplementary Table 1. Antibodies used

Name	Company	Dilution
Sorcin	Proteintech	WB 1:1000 IF 1:100
Sorcin	Abcam	IHC 1:500
STAT3	Proteintech	WB 1:1000 IF 1:100
p-STAT3	Proteintech	WB 1:1000
p-STAT3	CST	IHC 1:500
Insulin	Proteintech	IF 1:100
Insulin	Abcam	WB 1:1000 IHC 1:68000
GAPDH	Abcam	WB 1:10000
p38	HUABIO	WB 1:1000
p-p38	HUABIO	WB 1:1000
Actin	Proteintech	WB 1:10000
Tubulin	Proteintech	WB 1:10000
Pdx1	Abcam	IF 1:1000

Supplementary Table 2. Primer sequences used

Name	Primer Sequence (Forward 5' - 3')	Primer Sequence (Reverse 5' - 3')
GAPDH (human)	GCACCGTCAAGGCTGAGAAC	TGGTGAAGACGCCAGTGGA
STAT3 (human)	AACTCTCACGGACGAGGAGCT	AGTAGTGAACTGGACGCCGG
SRI (human)	TAAAGAACTCTGGGCTGTACTG	GTGCTGTATCGTTTTGCAATTG
IL8(human)	AACTGAGAGTGATTGAGAGTGG	ATGAATTCTCAGCCCTCTTCAA
CCL2(human)	ATGACTTCCAAGCTGGCCGTGGCT	ATGACTTCCAAGCTGGCCGTGGCT
CCL5(human)	GGCAGCCCTCGCTGTCATCCTCA	CTTGATGTGGGCACCGGGGCAGTG
SERPIN E1(human)	AACGTGGTTTTCTCACCCTAT	CAATCTTGAATCCCATAGCTGC
CXCL1(human)	AAGAACATCCAAAGTGTGAACG	CACTGTTCAGCATCTTTTCGAT
Gadph (mouse)	GGTGAAGGTCGGAGTCAACG	CAAAGTTGTCATGGATGHACC
Ins (mouse)	TTGTCAAACAGCATCTTTGTGG	GGACTTGGGTGTGTAGAAGAAG
Pdx1 (mouse)	GACATCTCCCCATACGAAGTG	GTGAGCTTTGGTGGATTTCATC
Mafa (mouse)	GCTTCAGCAAGGAGGAGGTCATC	GGCACTTCTCGCTCTCCAGAATG
Foxo1 (mouse)	AATTCACCCAGTCCAAACTACT	TACTGGTTCAATCCTCCGTAAC
Rfx6 (mouse)	ACATCTGCCATGAACCGCTTGAC	CACCCATCCTACCATAGCCATTGC

Supplementary Figures

Supplementary Figure 1

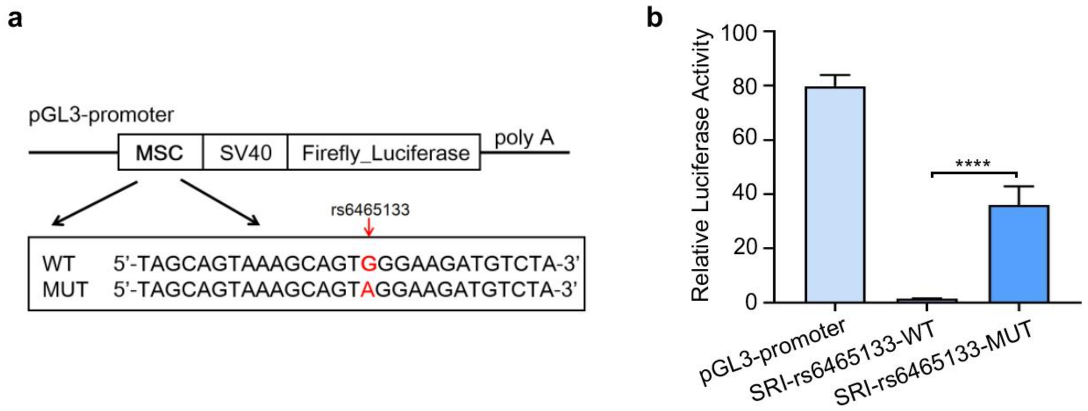

Supplementary Fig. 1. Point mutations of SNP rs6465133 differ in the transcriptional activity of *SRI* gene (a) The sequence of the firefly luciferase reporter plasmid in the dual-luciferase reporter gene experiment. (b) The relative luciferase activity in the dual-luciferase reporter gene experiment. Using Renilla luciferase as an internal control, the fluorescence intensity obtained from firefly luciferase was divided by the fluorescence intensity obtained from Renilla luciferase. pGL3-promoter was used as a negative control. ****P<0.0001. Statistical analysis was performed by Student's t-test analysis for two groups.

Supplementary Figure 2

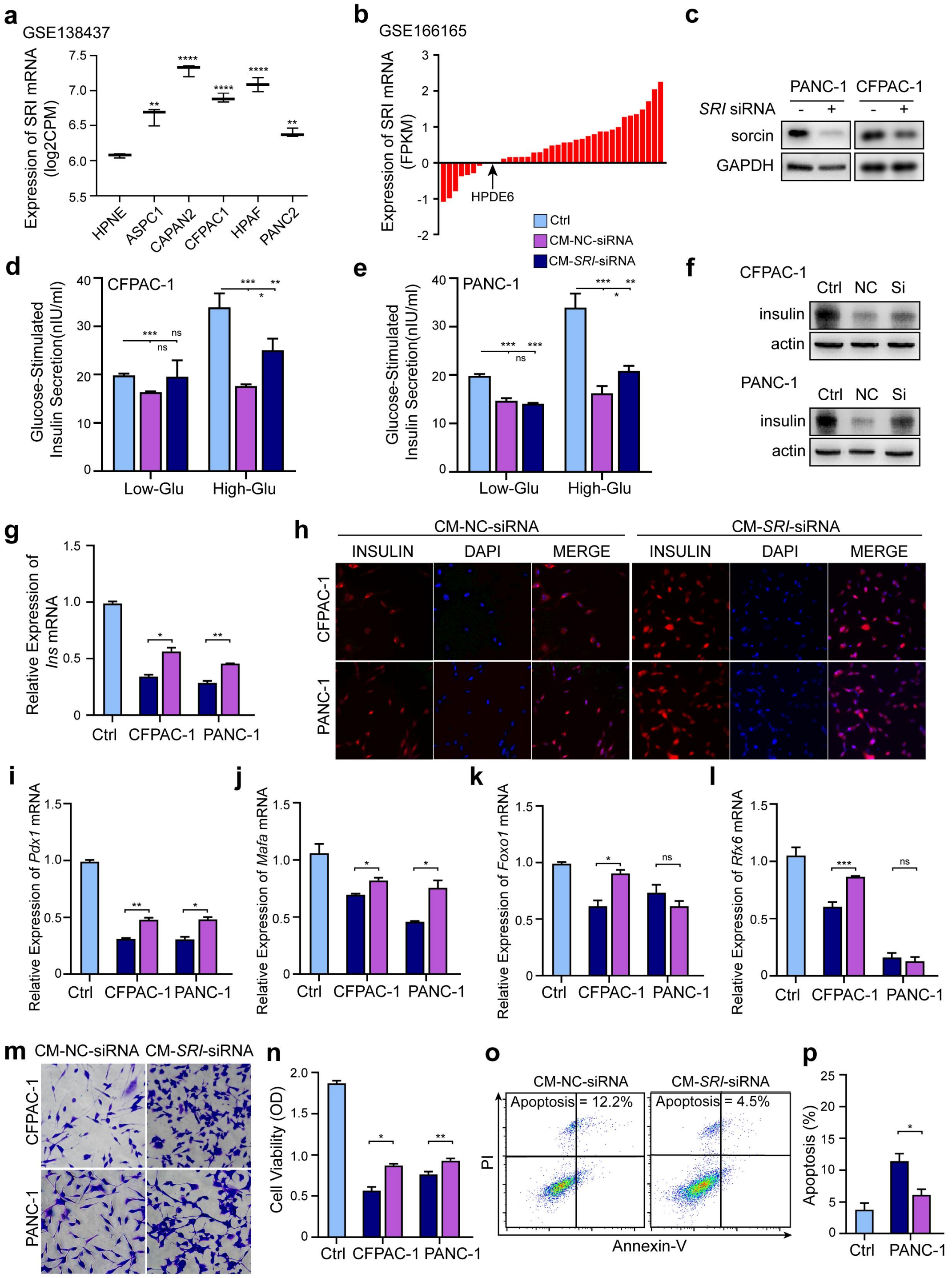

Supplementary Fig. 2. *In vitro* PC cells inhibit insulin secretion in MIN6 cells in a sorcin-dependent manner *SRI* expression in PC cell lines and normal pancreatic duct cell line (HPNE/ HPDE6) based on (a) GSE138437 dataset and (b) the GSE166165 dataset. (c) Detection of knockdown effect in PANC-1 and CFPAC-1 cells by immunoblotting. Insulin content in supernatant after GSIS in MIN6 cells incubated with conditioned medium form (d) PANC-1 and (e) CFPAC-1 cells pretreated with NC siRNA

(CM-NC-siRNA) and SRI siRNA (CM-*SRI*-siRNA). (f) Detection of insulin content in MIN6 cells incubated with CM-NC-siRNA and CM-*SRI*-siRNA from PANC-1 and CFPAC-1 cells by

immunoblotting. (g) The expression of *Ins* mRNA in MIN6 cells incubated with different conditioned medium from PANC-1 and CFPAC-1 cells. (h) Immunofluorescence shows the content of insulin in MIN6 cells treated by different conditioned medium from PANC-1 and CFPAC-1 cells. The expression of (i) *Pdx1*, (j) *Mafa*, (k) *Foxo1* and (l) *Rfx6* mRNA in MIN6 cells incubated with different conditioned medium from PANC-1 and CFPAC-1 cells. (m) Morphology of MIN6 cells treated with different conditioned medium from PANC-1 and CFPAC-1 cells. (n) Detection of cell viability of MIN6 cells treated with different conditioned medium from PANC-1 and CFPAC-1 cells by MTT assays. (o) Detection of apoptosis by flow cytometry in MIN6 treated with different conditioned medium from PANC-1. (p) Quantification results of apoptosis rate. Ns, no significance; *P<0.05; **P<0.01; ***P<0.001; ****P<0.0001, means ± SD was shown. Statistical analysis was performed by Student’s t-test analysis for two groups.

Supplementary Figure 3

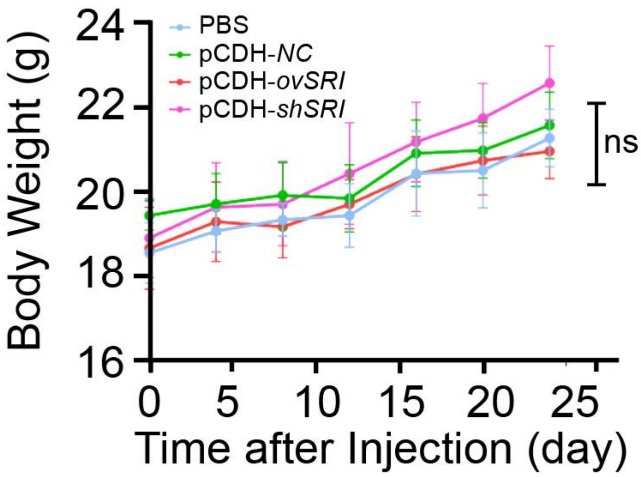

Supplementary Fig. 3. ***In vivo*** **PC inhibit insulin secretion in pancreas β-cells in a sorcin-dependent manner** Body weight monitoring was performed every 4 days during subcutaneous tumor formation in nude mice. Statistical analysis was performed by Student's t-test analysis for two groups and one-way ANOVA for multiple groups.

Supplementary Figure 4

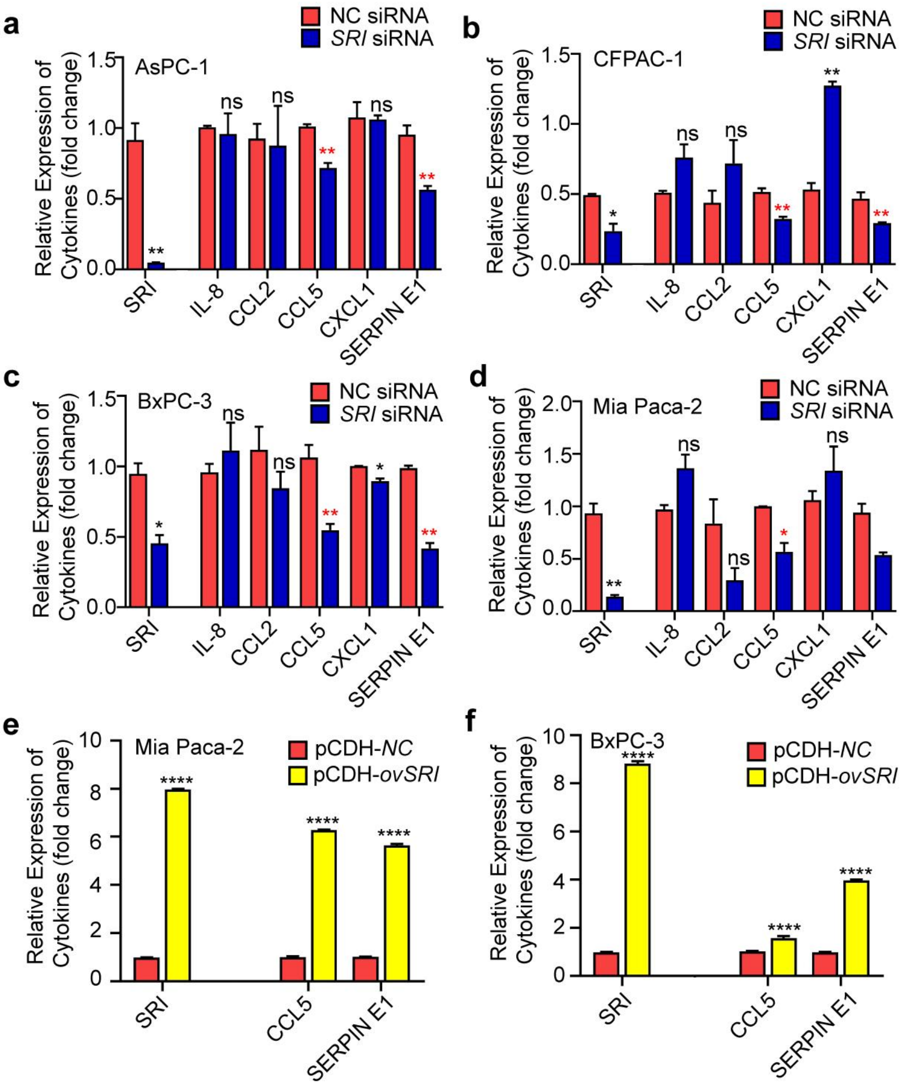

Supplementary Fig. 4. Sorcin-overexpressing PC cells release CCL5 and serpin E1 to inhibit insulin secretion in MIN6 cells

Detection of *SRI* and 5 down-regulated cytokines' mRNA by RT-PCR in (a) AsPC-1, (b) CFPAC-1, (c) BxPC-3 and (d) Mia Paca-2 after *SRI* knockdown. Detection of *SRI* , *CCL5* and *SERPIN E1* mRNA by RT-PCR in (e) Mia Paca-2 and (f) BxPC-3 after *SRI* over-expression. Ns, no significance; *P<0.05; **P<0.01; ***P<0.001; ****P<0.0001, means ± SD was shown. Statistical analysis was performed by Student's t-test analysis for two groups.

Supplementary Figure 5

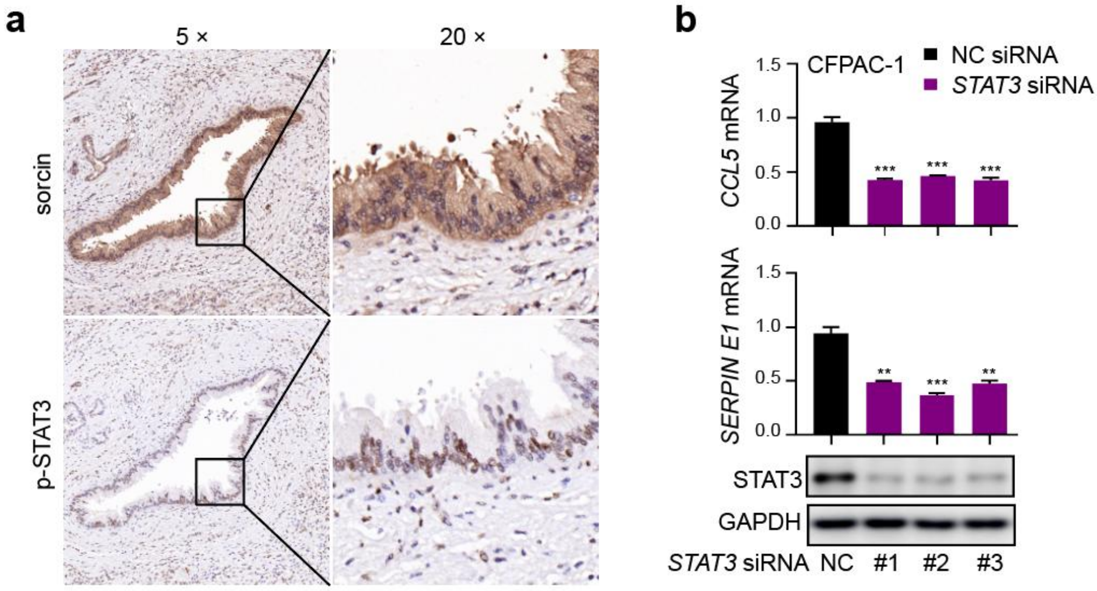

Supplementary Fig. 5. Sorcin up-regulates CCL5 and serpin E1 expression by forming a positive feedback loop with STAT3

(a) Immunohistochemistry staining shown that sorcin was highly expressed in cytoplasm, and p-STAT3 was aggregated in the nucleus in patients with pancreatic cancer. (b) Detection of *CCL5* and *SERPIN E1* mRNA, and the expression levels of STAT3 after treating with *STAT3* siRNA in CFPAC-1. **P<0.01; ***P<0.001, means ± SD was shown. Statistical analysis was performed by Student's t-test analysis for two groups.

Supplementary Figure 6

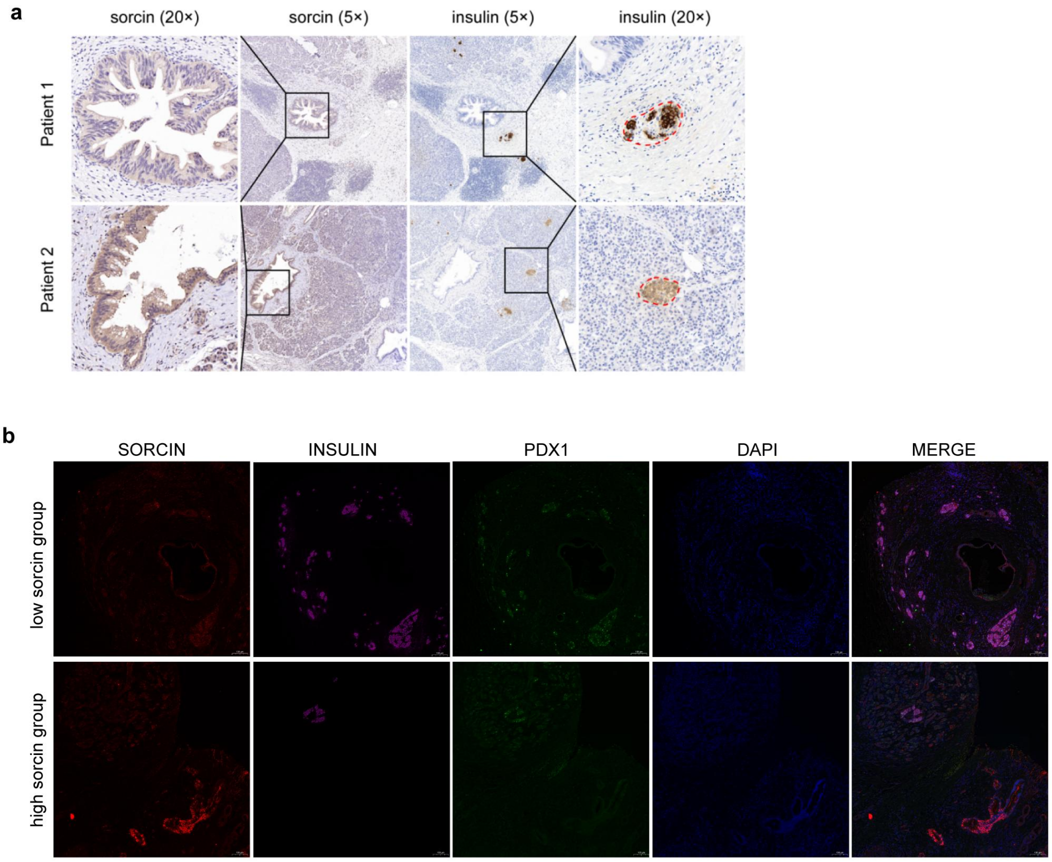

Supplementary Fig. 6. In the clinical cohort *SRI* can be used to differentiate PCAND from T2DM and downstream Serpin E1 may be the potential biomarker

(a) Negative correlation between the IHC intensity of sorcin in pancreatic cancer cells and IHC intensity of insulin in adjacent pancreatic islets. (b) Immunofluorescence of islets labeled with insulin and PDX1 proteins in the pancreas of PC patients with high or low sorcin levels.
